# Synthesis, Characterization and HPLC Analysis of the (1*S*,2*S*,5*R*)-Diastereomer and the Enantiomer of the Clinical Candidate AR-15512

**DOI:** 10.3390/molecules26040906

**Published:** 2021-02-09

**Authors:** Sergio Rodríguez-Arévalo, Eugènia Pujol, Sònia Abás, Carles Galdeano, Carmen Escolano, Santiago Vázquez

**Affiliations:** 1 Laboratori de Química Farmacèutica (Unitat Associada al CSIC), Facultat de Farmàcia i Ciències de l’Alimentació, Institute of Biomedicine (IBUB), Universitat de Barcelona, Av. Joan XXIII, 27-31, 08028 Barcelona, Spain; rodriguez.arevalo@ub.edu (S.R.-A.); epujol@ub.edu (E.P.); soniaabas88@gmail.com (S.A.); 2 Department of Pharmacy and Pharmaceutical Technology and Physical Chemistry, Faculty of Pharmacy and Food Sciences, Institute of Biomedicine (IBUB), University of Barcelona, Av. Joan XXIII, 27-31, 08028 Barcelona, Spain; cgaldeano@ub.edu

**Keywords:** absolute configuration, AR-15512, diastereomer, enantiomer, HPLC analytical method

## Abstract

AR-15512 (formerly known as AVX-012 and WS-12) is a TRPM8 receptor agonist currently in phase 2b clinical trials for the treatment of dry eye. This bioactive compound with menthol-like cooling activity has three stereogenic centers, and its final structure and absolute configuration, (1*R*,2*S*,5*R*), have been previously solved by cryo-electron microscopy. The route of synthesis of AR-15512 has also been reported, revealing that epimerization processes at the C-1 can occur at specific stages of the synthesis. In order to confirm that the desired configuration of AR-15512 does not change throughout the process and to discard the presence of the enantiomer in the final product due to possible contamination of the initial starting material, both the enantiomer of AR-15512 and the diastereomer at the C-1 were synthesized and fully characterized. In addition, the absolute configuration of the (1*S*,2*S*,5*R*)-diastereomer was determined by X-ray crystallographic analysis, and new HPLC methods were designed and developed for the identification of the two stereoisomers and their comparison with the clinical candidate AR-15512.

## 1. Introduction

AR-15512 ((1*R*,2*S*,5*R*)-*N*-(4-methoxyphenyl)-5-methyl-2-(1-methylethyl) cyclohexanecarboxamide) is a bioactive compound with menthol-like cooling effects that is currently being evaluated in phase 2b clinical trials as an ophthalmic solution for the treatment of dry eye, a chronic, highly prevalent, and age-related condition associated with pain and limitations in performing daily activities [1,2,3,4]. Indeed, dry eye disease is nowadays considered an unmet medical need, causing a significant socioeconomic burden in the general population [5,6]. AR-15512 acts as a potent and selective agonist of the TRPM8 (transient receptor potential melastatin member 8) cold thermoreceptor. TRPM8 is a calcium-permeable ion channel that serves as the principal detector of cold in humans and is involved in the regulation of tear production and blink rate [7,8]. Importantly, this stimulation can ultimately lead to a restoration of tear film volume and a reduction of ocular discomfort in patients suffering from dry eye syndrome [4]. AR-15512, formerly known as AVX-012, was developed by Avizorex Pharma, S. L. (Barcelona, Spain), a Spanish ophthalmic pharmaceutical company working on new therapeutics for tackling dry eye syndrome [9]. In November 2019, Avizorex and AR-15512 were acquired by Aerie Pharmaceuticals (Durham, NC, USA), a company that has further progressed this clinical candidate from successful phase 2a studies performed by Avizorex to the aforementioned phase 2b, with a topline readout expected in the third quarter of 2021 [10,11]. Curiously, this molecule was first described in the literature in 1971, when it was patented as a menthol-derived cooling agent under the name of WS-12 [12]. Since then, other patents and several published works have followed, including studies confirming its exact stereochemistry and its binding to the TRPM8 receptor [13].

**Scheme 1:**
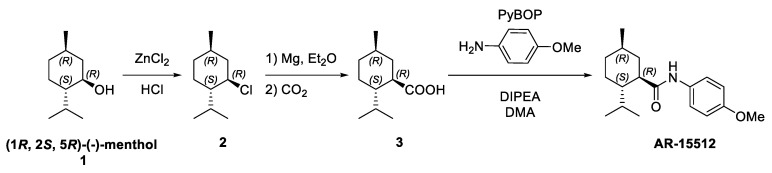
Structure of the product AR-15512 and its reported synthetic procedure.

AR-15512 is easily synthesized starting from (1*R*,2*S*,5*R*)-(−)-menthol (**1**), a small molecule terpenoid from peppermint oil, as previously reported (Scheme 1) [12,14,15]. Hence, the first step is the reaction of the commercially available **1** with zinc chloride in the presence of hydrochloric acid to give (1*S*,2*R*,4*R*)-2-chloro-1-isopropyl-4-methylcyclohexane (**2**). Then, treatment of this intermediate with magnesium in diethyl ether to prepare the Grignard reagent followed by in situ reaction with CO_2_ provides the corresponding carboxylic acid (**3**). Finally, a reaction between **3** and *p*-anisidine in the presence of the coupling reagent benzotriazol-1-yl-oxytripyrrolidinophosphonium hexafluorophosphate (PyBOP), *N*,*N*-diisopropylethylamine (DIPEA) as a base and dimethylacetamide (DMA) as a solvent furnishes AR-15512. Alternatively, amide formation can also be achieved via the corresponding acid chloride of **3**, readily prepared by reaction with SOCl_2_ and like reagents, which reacts with the same *p*-anisidine in appropriate base and solvent.

**Figure 1:**
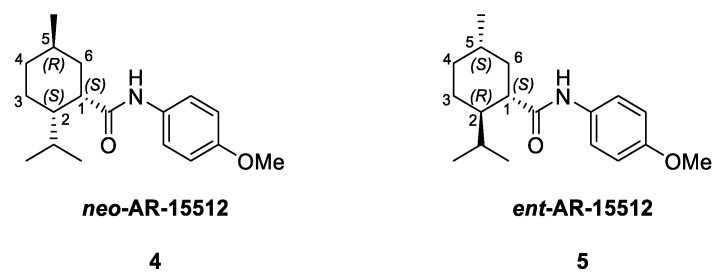
Structure and configuration of the target compounds in this work.

Bearing in mind both the chemical structure of AR-15512 and its synthesis, it has not been fully discarded whether epimerization processes at the C-1 can take place during its obtention [16]. Therefore, in order to rule this out and to confirm that the synthetic sequence indicated in Scheme 1 is stereochemically robust, the synthesis of the (1*S*,2*S*,5*R*)-diastereoisomer of AR-15512 (*neo*-AR-15512, **4**) was envisaged. Furthermore, in order to discard the presence of the enantiomer of AR-15512 (*ent*-AR-15512, **5**) in the final product coming from possible contamination of the initial (1*R*,2*S*,5*R*)-(−)menthol with its enantiomer (1*S*,2*R*,5*S*)-(+)menthol, a synthetic sequence was developed to access to the enantiomer of AR-15512 (Figure 1).

Here, we report for the first time the synthesis and full characterization of both the (1*S*,2*S*,5*R*)-diastereoisomer, **4**, and the enantiomer of AR-15512, **5**. In addition, we provide detailed information on the high-performance liquid chromatography (HPLC) analytical methods that have been developed for the identification of both compounds and comparison with the clinical candidate. Finally, X-ray crystallographic analysis has been performed to confirm the absolute configuration of the (1*S*,2*S*,5*R*)-diastereoisomer **4**.

## 2. Results and Discussion

### 2.1. Synthesis of (1S,2S,5R)-diastereomer of AR-15512, neo-AR-15512, ***4***

**Scheme 2:**
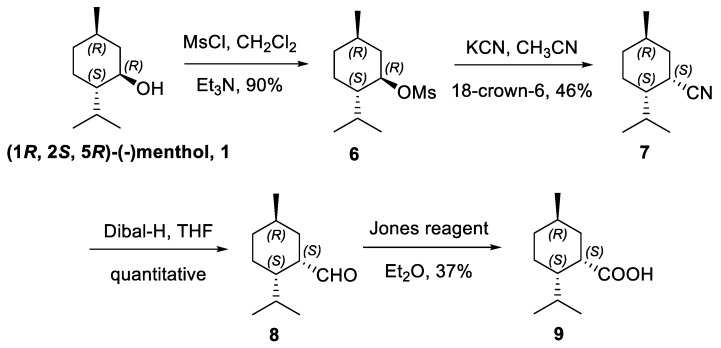
Synthetic procedure reported for the carboxylic acid **9** [17].

As previously mentioned, the synthetic sequence depicted in Scheme 1 to approach AR-15512 starts from the commercially available (1*R*,2*S*,5*R*)-(−)-menthol (**1**). In order to access to (1*S*,2*S*,5*R*)-*N*-(4-methoxyphenyl)-5-methyl-2-(1-methylethyl)cyclohexanecarboxamide (*neo*-AR-15512) **4**, the diastereoisomer of AR-15512 at the C-1, an inversion in the configuration of the stereocenter C-1 is required from the same starting material. For its obtention, we took advantage of the fact that the synthesis of the carboxylic acid derivative (**9**) had already been published (Scheme 2) [17]. Worth to highlight, in this reference, the author reported that the aldehyde (**8**) was prone to epimerization.

**Scheme 3:**
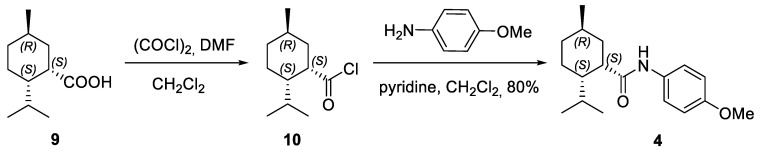
Synthesis of *neo*-AR-15512, **4**, from the known carboxylic acid **9**.

Hence, starting from the known compound **9,** the corresponding acid chloride (**10**) was prepared by treatment with oxalyl chloride in dry dichloromethane (DCM) and in the presence of a catalytic amount of dimethylformamide (DMF) under argon. Intermediate **10** was used without further purification into the next synthetic step. Finally, the formation of compound **4** was achieved in 80% overall yield by reaction of **10** with *p*-anisidine in dry DCM as a solvent and in the presence of pyridine as a base (Scheme 3).

#### 2.1.1. Characterization and X-ray Structure Analysis of **4**

**Figure 2:**
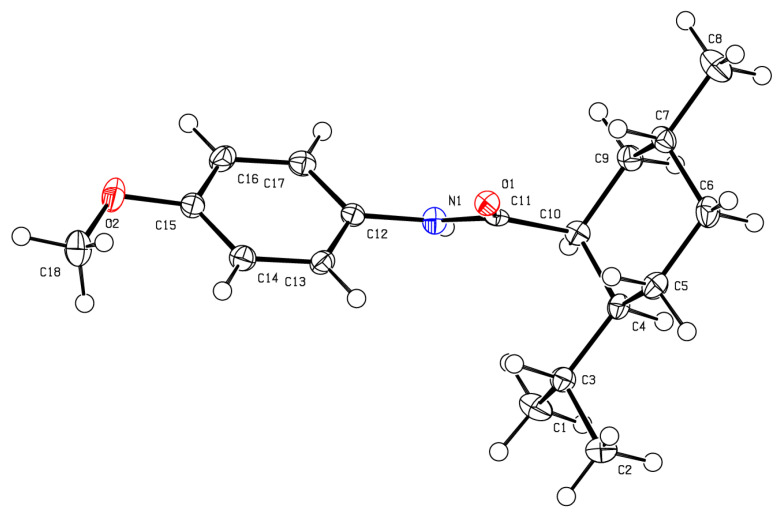
Crystal structure for (1*S*,2*S*,5*R*)-diastereomer of AR-15512, **4**.

The diastereoisomer of AR-15512 at the C-1, compound **4**, was completely characterized by means of ^1^H, ^13^C, COSY and HSQC NMR experiments, infrared, elemental analysis, specific optical rotation, and melting point (see Material and Methods section and Supplementary Materials for further details). Moreover, the chiral identity of its three stereogenic centers was unambiguously confirmed by X-ray crystallography of a monocrystal obtained by recrystallization from ethyl acetate (Figure 2). Compound **4** was found to crystallize in the orthorhombic system, space group P 21 21 21, with the following cell parameters: *a* = 9.1371(2) Å, *b* = 10.3821(3) Å, *c* = 17.4893(7) Å, V = 1659.98(7) Å^3^ and Z = 4 (further crystal data can be found in Supplementary Materials) [18].

#### 2.1.2. HPLC Method for the Analysis of AR-15512 and Its Diastereoisomer **4** (*neo*-AR-15512)

Importantly, once the diastereoisomer of AR-15512 at the C-1 was synthesized, an HPLC method for its identification and differentiation from AR-15512 was designed and developed. Thus, AR-15512 and **4** were distinguished by comparison of the retention times of both compounds separately and in a mixture by using an Ultrabase C-18 (Akady) 5 ODS column 250 mm × 4.6 mm (particle size 5 µM) as stationary phase and an acetonitrile, water and trifluoroacetic acid (TFA) (0.1%) as a mobile phase, with a UV detector set at 254 nm (Table 1) (see Materials and Methods and Supplementary Materials for further details).

### 2.2. Synthesis of the Enantiomer of AR-15512, **5** (ent-AR-15512)

In pursuance of the enantiomer of AR-15512, (1*S*,2*R*,5*S*)-*N*-(4-methoxyphenyl)-5-methyl-2-(1-methylethyl)cyclohexanecarboxamide **5**, we first considered following the synthetic route previously shown in Scheme 1, but starting from the corresponding enantiomer, i.e., (1*S*,2*R*,5*S*)-(+)-menthol. However, an interesting alternative was found based on the synthetic accessibility of the epimerization-prone aldehyde **8**, which had indeed been used for obtaining diastereomer **4**, according to the bibliographic reference (see Scheme 2) [17].

**Scheme 4:**
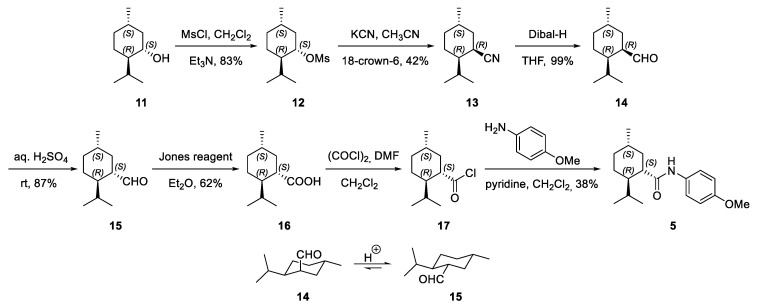
Route of synthesis for **5**, *ent*-AR-15512, and key epimerization of **14** to **15**.

Therefore, considering the known lability of the hydrogen in the α-position of the aldehyde derivative **8**, we reasoned that following the same sequence depicted in Scheme 2, but starting from the enantiomer of **1**, (1*S*,2*R*,5*S*)-(+)-menthol (**11**), we may gain access to the enantiomer of **8**, the aldehyde (**14**). We hypothesized that epimerization at C-1 of the intermediate aldehyde **14** should take place under acidic conditions as it would produce the thermodynamically more stable diastereoisomer **15**, in which all three substituents (aldehyde, isopropyl and methyl) are in equatorial (Scheme 4). The latter would then serve as an intermediate for the synthesis of the final compound **5**.

To our delight, the envisaged synthetic strategy turned out to be successful and allowed us to synthesize **5**. In this manner, the first synthetic step involved the treatment of **11** with mesyl chloride in dry DCM and in the presence of triethylamine as a base to yield the mesylate derivative (**12**) in 83% yield. In the next step, the inversion of the configuration in the C-1 occurred by means of an S_N_2 reaction of the mesylate group by cyanide. The reaction of mesylate **12** with potassium cyanide in dry acetonitrile and in the presence of equimolecular quantities of 18-crown-6 furnished the nitrile derivative (**13**) as a unique product with an *R* configuration in the C-1 and in 42% yield. The following step involved the reduction of the nitrile to the aldehyde **14** in nearly quantitative yield with DIBAL-H in dry THF. As expected, the aldehyde **14** is susceptible to epimerization by treatment with aqueous sulfuric acid furnishing in 87% yield its epimer **15** that has the required stereochemistry in the three stereogenic centers to access the enantiomer of AR-15512. Oxidation of **15** using the Jones reagent yielded carboxylic acid **16** in 62% yield. Subsequently, treatment of **16** with oxalyl chloride in dry DCM and in the presence of a catalytic amount of DMF provided acid chloride (**17**), which was then reacted with *p*-anisidine in DCM under basic conditions to give the final compound, enantiomer **5**, with 38% yield (Scheme 4).

#### 2.2.1. Characterization of **5**

The final compound **5** was fully characterized by ^1^H, ^13^C, COSY and HSQC NMR experiments, infrared, elemental analysis, specific optical rotation, and melting point (see Material and Methods section and Supplementary Materials for further details).

#### 2.2.2. Chiral HPLC Method for the Analysis of AR-15512 and Its Enantiomer 5 (*ent*-AR-15512)

Just as we proceeded with the diastereomer **4**, a specific HPLC method was designed and developed to demonstrate that compound **5** was indeed the enantiomer of the clinical candidate AR-15512 and to discern between them. In particular, the chiral HPLC method was set up using a chiral column (Chiralpack IC 250 mm × 4.6 mm (particle size 5 µM)) as stationary phase and a mixture of hexane and 2-propanol as mobile phase, with a UV detector set at 254 nm (for detailed information, see Materials and Methods and Supplementary Materials). The retention times of the two compounds are shown below (Table 2).

## 3. Materials and Methods

### 3.1. Chemical Synthesis

#### 3.1.1. General Methods

Commercially available reagents and solvents were used without further purification unless stated otherwise. Column chromatography was performed on silica gel 60 (35–70 μm) with the indicated solvent system. Thin-layer chromatography was performed with aluminum-backed sheets with silica gel 60 F_254_ (Merck, ref 1.05554), and spots were visualized with UV light and 1% aqueous solution of KMnO_4_. Melting points were determined in open capillary tubes with an MFB 595010M Gallenkamp instrument. IR spectra were performed in a Shimadzu IRAffinity-1S spectrophotometer, and only noteworthy IR absorptions (cm^−1^) are listed. Optical rotations were measured on a PerkinElmer 341 polarimeter. NMR spectra were recorded in CDCl_3_ at 400 MHz (^1^H) and 100.6 MHz (^13^C). Chemical shifts are reported in δ values downfield from TMS or relative to residual chloroform (7.26 ppm, 77.0 ppm) as an internal standard. Data are reported in the following manner: chemical shift, multiplicity, coupling constants (*J*) in hertz (Hz), integrated intensity, and assignment. Multiplicities are reported using the following abbreviations: s, singlet; dd, doublet of doublets; ddd, doublet of a doublet of doublets; dddd, doublet of a doublet of a doublet of doublets; m, multiplet; dm, double multiplet; br s, broad signal. Assignments given for the NMR spectra of the compounds were carried out on the basis of COSY ^1^H/^1^H (standard procedures) and COSY ^1^H/^13^C (gHSQC) experiments. The elemental analyses were carried out in a Flash 1112 series ThermoFinnigan elemental microanalyzer (A5) to determine C, H, and N. The two final compounds possessed purity ≥95%, as evidenced by their elemental analyses.

#### 3.1.2. Synthesis of Intermediates **6**–**9**

Compounds **6**, **7**, **8** and **9** were synthesized according to previously published procedures [17].

#### 3.1.3. Synthesis of (1*S*,2*S*,5*R*)-5-methyl-2-(1-methylethyl)cyclohexanecarbonyl Chloride, **10**

The carboxylic acid **9** (293 mg, 1.6 mmol) and a catalytic amount of dry DMF (6 µL, 0.08 mmol) were put in dry DCM (10 mL) under argon and cooled to 0 °C, followed by the slow addition of oxalyl chloride (0.17 mL, 1.9 mmol) over a 10 min period. When the reaction was completed (monitored by NMR), the solvent was removed in vacuo. A yellow oil was obtained (323 mg, 100%) and was used in the next step without further purification. Its ^1^H NMR spectrum was in agreement with the reported data [19].

#### 3.1.4. Synthesis of neo-AR-15512, (1*S*,2*S*,5*R*)-*N*-(4-methoxyphenyl)-5-methyl-2-(1-methylethyl)cyclohexanecarboxamide, **4**

To a solution of *p*-anisidine (296 mg, 2.4 mmol) in dry DCM (8 mL) under argon, pyridine (0.2 mL, 2.4 mmol) was added. The solution was cooled to 0 °C and treated with the previously obtained compound **10** (323 mg, 1 mmol) dissolved in DCM (2 mL). The reaction mixture was stirred at room temperature overnight. The mixture was filtered, and the resulting filtrate was treated with 5 N aqueous NaOH until pH = 14 and subsequently extracted with DCM (3 × 50 mL). The combined organic extracts were dried over anhydrous Na_2_SO_4_, filtered and concentrated under reduced pressure to provide a yellow solid. Purification by column chromatography on silica with 100% DCM as eluent gave pure compound **4** as white crystals (360 mg, 78%), mp (ethyl acetate) 152 °C (151 °C [19]). [α]^22^_D_ = +17.6 (*c* 0.23, EtOH) ([α]^22^_D_ = +16.4 (*c* 1.0, EtOH) [19]). IR (KBr) ν: 527, 679, 822, 826, 1036, 1165, 1246, 1510, 1520, 1643, 1651, 2920, 2938, 2949, 3289, 3412, 3480 cm^−1^. ^1^H NMR (400 MHz, COSY, HETCOR, CDCl_3_), δ: 0.84 (d, *J* = 6.4 Hz, 3 H, 5C-CH_3_), 0.90 (m, 1 H, 4-H_ax_), 0.91 [d, *J* = 6.4 Hz, 3 H, CH(CH_3_)_2_], 0.94 [d, *J* = 6.8 Hz, 3 H, CH(CH_3_)_2_], 1.08 (ddd, *J* = 16.8 Hz, *J*’ = 8.0 Hz, *J*’’ = 4.0 Hz, 1 H, 2-H), 1.25 (m, 1 H, 6-H_ax_), 1.67–1.73 (m, 2 H, 3-H_a_, 5-H), 1.81 (dm, *J* = 12.8 Hz, 1 H, 4-H_eq_), 1.89–1.99 [m, 3 H, 3-H_b_, 6-H_eq_, CH(CH_3_)_2_], 2.67 (br s, 1 H, 1-H), 3.78 (s, 3 H, OCH_3_), 6.84 [m, 2 H, 3(5)-ArH], 7.04 (br s, 1 H, NH), 7.39 [m, 2 H, 2(6)-ArH]. ^13^C NMR (100.6 MHz, CDCl_3_), δ: 21.3 [CH_3_, CH(CH_3_)_2_], 21.7 [CH_3_, CH(CH_3_)_2_], 22.5 (CH_3_, 5C-CH_3_), 25.5 (CH_2_, 3-C), 27.1 [CH, CH(CH_3_)_2_], 30.5 (CH, 5-C), 35.3 (CH_2_, 4-C), 39.3 (CH_2_, 6-C), 44.3 (CH, 1-C), 46.8 (CH, 2-C), 55.3 (CH_3_, OCH_3_), 114.1 [CH, 3(5)-ArC], 121.7 [CH, 2(6)-ArC], 131.3 (C, 1-ArC), 156.2 (C, 4-ArC), 173.4 (C, CON). Anal. calcd for C_18_H_27_NO_2_: C 74.70, H 9.40, N 4.84. Found: C 74.90, H 9.34, N 4.69.

#### 3.1.5. Synthesis of (1*S*,2*R*,5*S*)-(+)-menthol Mesylate, **12**

A solution of (1*S*,2*R*,5*S*)-(+)-menthol (**11**) (15 g, 96 mmol) in dry DCM (188 mL) under argon was cooled to 0 °C, and to that methane sulfonyl chloride (9.3 mL, 120 mmol) was added in one portion. The mixture was stirred for 20 min, followed by a dropwise addition of triethylamine (17.5 mL, 124.8 mmol) to the cold solution. The mixture was stirred for 24 h at 20 °C, whereupon complete consumption of starting material was shown by TLC analysis on SiO_2_ using 15% ethyl acetate in hexane as eluent. The mixture was diluted with DCM (200 mL) and washed with water (2 × 100 mL), saturated aqueous NaHCO_3_ (100 mL) and saturated aqueous NaCl (50 mL). The organic solution was dried over anhydrous Na_2_SO_4_, filtered, and the solvent removed in vacuo. The pale yellow oil was chromatographed on silica using 10% ethyl acetate in hexane as eluent to give **12** as a colorless oil (18.6 g, 83%). Its ^1^H NMR spectrum was in agreement with the reported data for ent-**12** [17]. ^1^H (400 MHz, CDCl_3_), δ: 0.83 (d, *J* = 6.8 Hz, 3 H, 5C-CH_3_), 0.87 (m, 1 H, 4-H_ax_), 0.92 [d, *J* = 6.8 Hz, 3 H, CH(CH_3_)_2_], 0.93 [d, *J* = 6.8 Hz, 3 H, CH(CH_3_)_2_], 1.04 (m, 1 H), 1.20 (m, 1 H), 1.30–1.49 (complex signal, 2 H), 1.57–1.69 (complex signal, 2 H), 2.00 (m, 1 H), 2.19 (dm, *J* = 12.0 Hz, 1 H), 3.00 (s, 3 H, SO_2_CH_3_), 4.54 (ddd, *J* = 10.8 Hz, *J′* = 10.8 Hz, *J″* = 4.4 Hz, 1 H, 1-H).

#### 3.1.6. Synthesis of (1*R*,2*R*,5*S*)-2-isopropyl-5-methylcyclohexane-1-carbonitrile, **13**

To a solution of the mesylate **12** (5.76 g, 24.6 mmol) in dry acetonitrile (70 mL) under argon was added potassium cyanide (8 g, 123 mmol) and 18-crown-6 (6.5 g, 24.6 mmol) and the resulting mixture was heated to reflux for 48 h. The mixture was poured into DCM (100 mL). The yellow solution was washed with water (5 × 30 mL) to remove excess cyanide and 18-crown-6. The organic phase was washed with saturated aqueous NaCl (50 mL) and dried over anhydrous Na_2_SO_4_. The solution was filtered, and the solvents were removed in vacuo. The resulting orange oil was chromatographed on silica with 5% ethyl acetate in hexane as eluent, affording compound **13** as a pale yellow oil (1.68 g, 42%). Its ^1^H NMR spectrum was in agreement with the reported data for ent-**13** [20]. ^1^H (400 MHz, CDCl_3_), δ: 0.85 (m, 1 H), 0.92 (d, *J* = 6.8 Hz, 3 H, 5C-CH_3_), 0.96 [d, *J* = 6.4 Hz, 6 H, CH(CH_3_)_2_], 1.00 (m, 1 H), 1.15 (ddd, *J* = 13.2 Hz, *J*’ = 12.0 Hz, *J*’’ = 4.0 Hz, 1 H), 1.28 (m, 1 H), 1.59 (m, 1 H), 1.68–1.82 (complex signal, 2 H), 1.89 (m, 1 H), 1.99 (dq, *J* = 13.2 Hz, *J*’ = 2.8 Hz, 1 H), 3.06 (m, 1 H, 1-H).

#### 3.1.7. Synthesis of (1*R*,2*R*,5*S*)-2-isopropyl-5-methylcyclohexane-1-carboxaldehyde, **14**

A solution of nitrile **13** (1.41 g, 8.5 mmol) in dry THF (10 mL) under argon was cooled to 0 °C. Diisobutylaluminum hydride (17.5 mL, 1.2 M in toluene, 21.3 mmol) was added dropwise. The resulting solution was stirred at 0 °C for 4 h, then allowed to warm to 25 °C, and stirred for 18 additional hours. To quench, 200 mL of diethyl ether and 100 mL of 2% aqueous H_2_SO_4_ were cooled to 0 °C. The reaction mixture was added in 5 mL aliquots to the two-phase mixture with stirring between additions. After complete addition, the two-phase mixture was further acidified with 4 mL of 2% aqueous H_2_SO_4_. The organic phase was separated, and the aqueous phase was extracted with diethyl ether (3 × 150 mL). The organic phases were combined and washed with saturated aqueous NaCl (100 mL), then dried over anhydrous Na_2_SO_4_, filtered, and the solvents removed in vacuo. **14** was isolated as a pale yellow oil (1.41 g, 99%). Due to its instability upon chromatography, this compound was used without further purification. Its ^1^H NMR was in agreement with the reported data for *ent*-**14** [17].

#### 3.1.8. Synthesis of (1*S*,2*R*,5*S*)-2-isopropyl-5-methylcyclohexane-1-carboxaldehyde, **15**

A solution of aldehyde **14** (0.84 g, 5 mmol) in diethyl ether (50 mL) was cooled to 0 °C, followed by a dropwise addition of 25% aqueous H_2_SO_4_ (18 mL) to the cold solution. The resulting solution was stirred at 0 °C for 4 h and at room temperature overnight. The organic phase was separated, and the aqueous phase was extracted with diethyl ether (3 × 100 mL). The organic phases were combined and washed with saturated aqueous NaCl (100 mL), then dried over anhydrous Na_2_SO_4_, filtered, and the solvents removed in vacuo. Aldehyde **15** was isolated as a pale orange oil (732 mg, 87%). Due to its instability upon chromatography, **15** was used in the next step without further purification. Its ^1^H NMR was in agreement with previously reported data [21].

#### 3.1.9. Synthesis of (1*S*,2*R*,5*S*)-2-isopropyl-5-methylcyclohexane-1-carboxylic Acid, **16**

To a solution of the aldehyde **15** (732 mg, 4.35 mmol) in 30 mL of diethyl ether and cooled to 0 °C, whereupon an excess of Jones reagent (8 mL) was transferred dropwise via pipet until the reaction solution remained orange colored. This mixture was vigorously stirred at room temperature for 60 min. A few drops of 2-propanol were then added until the mixture remained dark green colored and the solvents were removed under reduced pressure. The green solid residue was dissolved in water (50 mL), and this mixture was extracted with diethyl ether (4 × 50 mL). The combined organic layers were concentrated under reduced pressure until about 50 mL of the solvent remained. Then, it was washed with 2 N aqueous NaOH (3 × 50 mL), the combined basic layers acidified at 0 °C with conc. HCl until the pH was adjusted to about 2. The white precipitate thus formed was extracted with diethyl ether (3 × 40 mL); the combined ethereal layers were successively washed with one portion of water and saturated aqueous NaCl and dried over anhydrous Na_2_SO_4_. The dried ethereal solution was filtered through Celite^®^ to remove any remaining chromium impurities, and the solvent was then removed in vacuo to give yellow crystals of acid **16** (500 mg, 62%). Its ^1^H NMR was in agreement with previously reported data [22].

#### 3.1.10. Synthesis of (1*S*,2*R*,5*S*)-2-isopropyl-5-methylcyclohexane-1-carbonyl Chloride, **17**

The carboxylic acid **16** (0.45 g, 2.44 mmol) and a catalytic amount of dry DMF (9 µL, 0.12 mmol) were put in dry DCM (15 mL) under argon and cooled to 0 °C, followed by the slow addition of oxalyl chloride (0.25 mL, 2.93 mmol) over a 10 min period. When the reaction was completed (monitored by NMR), the solvent was removed in vacuo. Compound **17** was obtained as a yellow oil (493 mg, 100%). Its ^1^H NMR was in agreement with the reported data for ent-**17** [19].

#### 3.1.11. Synthesis of ent-AR-15512, (1*S*,2*R*,5*S*)-*N*-(4-methoxyphenyl)-5-methyl-2-(1-methylethyl)cyclohexanecarboxamide, **5**

To a solution of the *p*-anisidine (451 mg, 3.66 mmol) in dry DCM (16 mL) under argon, pyridine (0.3 mL, 3.66 mmol) was added. The solution was then cooled to 0 °C and treated with the corresponding acid chloride **17** (493 mg, 2.44 mmol) dissolved in DCM (4 mL). The reaction mixture was stirred at room temperature overnight. The mixture was filtered, and the resulting filtrate treated with 5 N aqueous NaOH until pH = 14 and subsequently extracted with DCM (3 × 50 mL). The combined organic extracts were dried over anhydrous Na_2_SO_4_, filtered and concentrated under reduced pressure to provide a yellow solid. The resulting solid was chromatographed on silica with 100% DCM as eluent. The final compound **5** was obtained as white crystals (245 mg, 38%), mp (ethyl acetate) 178.6 °C. [α]^22^_D_ = +56.0 (*c* 0.25, EtOH).

The specific optical rotation of AR-15512 was also measured [α]^22^_D_ = −57.6 (*c* 0.25, EtOH) ([α]^22^_D_ = −58.6 (*c* 1.0, EtOH) [19]). IR (KBr) ν: 725, 833, 1032, 1165, 1236, 1302, 1510, 1539, 1645, 1651, 2909, 2967, 3044, 3231 cm^−1^. ^1^H (400 MHz, COSY, HETCOR, CDCl_3_), δ: 0.83 (d, *J* = 6.8 Hz, 3 H, 5C-CH_3_), 0.91 [d, *J* = 6.0 Hz, 3 H, CH(CH_3_)_2_], 0.92 [d, *J* = 6.8 Hz, 3 H, CH(CH_3_)_2_], 0.99 (m, 1 H, 4-H_ax_), 1.07 (m, 1 H, 3-H_b_), 1.29 (m, 1 H, 6-H_ax_), 1.30 (m, 1 H, 5-H), 1.61 (dddd, *J* = 11.2 Hz, *J*’ = 11.2 Hz, *J″*= 2.8 Hz, *J*‴ = 2.8 Hz, 1 H, 2-H), 1.70 (m, 1 H, 3-H), 1.75 (m, 1 H, 4-H_eq_), 1.82 [m, 1 H, CH(CH_3_)_2_], 1.87 (dm, *J* = 11.6 Hz, 1 H, 6-H_eq_), 2.12 (ddd, *J* = 11.2 Hz, *J*’ = 11.2 Hz, *J*’’ = 3.2 Hz, 1 H, 1-H), 3.78 (s, 3 H, OCH_3_), 6.84 [m, 2 H, 3(5)-ArH], 7.12 (br s, 1 H, NH), 7.42 [m, 2 H, 2(6)-ArH]. ^13^C NMR (100.6 MHz, CDCl_3_), δ: 16.3 (CH_3_, 5C-CH_3_), 21.4 [CH_3_, CH(CH_3_)_2_], 22.3 [CH_3_, CH(CH_3_)_2_], 24.0 (CH_2_, 3-C), 28.8 [CH, CH(CH_3_)_2_], 32.3 (CH, 5-C), 34.5 (CH_2_, 4-C), 39.4 (CH_2_, 6-C), 44.6 (CH, 2-C), 50.6 (CH, 1-C), 55.5 (CH_3_, OCH_3_), 114.1 [CH, 3(5)-ArC], 121.7 [CH, 2(6)-ArC], 131.1 (C, 1-ArC), 156.3 (C, 4-ArC), 174.0 (C, CON). Anal. calcd for C_18_H_27_NO_2_: C 74.70, H 9.40, N 4.84. Found: C 74.62, H 9.26, N 4.64.

### 3.2. X-ray Crystallographic Analysis of neo-AR-15512, ***4***

A colorless needle-like specimen of C_18_H_27_NO_2_ (M = 289.42 g/mol), compound **4**, approximate dimensions 0.046 mm × 0.101 mm × 0.576 mm, was used for the X-ray crystallographic analysis. The X-ray intensity data were measured with a D8 Venture system equipped with a multilayer monochromator and a Cu microfocus (λ = 1.54178 Å) at 100.2 K. The frames were integrated with the Bruker SAINT software package using a narrow-frame algorithm. The structure was solved and refined using the Bruker SHELXTL Software Package, using the space group P 21 21 21, with Z = 4 for the formula unit C_18_H_27_NO_2_ (orthorhombic system). The final cell constants of *a* = 9.1371(2) Å, *b* = 10.3821(3) Å, *c* = 17.4893(7) Å, V = 1659.98(7) Å^3^, are based upon the refinement of the XYZ-centroids of reflections above 20 σ(I). Data were corrected for absorption effects using the multiscan method (SADABS). For further information, refer to Supplementary Materials [18].

### 3.3. HPLC Equipment and Methods

#### 3.3.1. HPLC Equipment

All analyses were carried out with a Waters HPLC system with a 1525 binary pump coupled to a UV detector Waters 2489 set at 254 nm and using Waters Breeze software.

#### 3.3.2. HPLC Method for Identification of AR-15512 and *neo*-AR-15512, **4**

*Sample concentration*. Solid sample 1 mg AR-15512 in 1 mL acetonitrile; solid sample 1 mg compound **4** in 1 mL acetonitrile; and solid sample mixture 1 mg AR-15512/1 mg compound **4** in 2 mL acetonitrile.

*Chromatographic conditions*. The employed column was a Ultrabase C-18 (Akady) 5 ODS 4.6 mm × 250 mm (5 μm), with a constant flow of 1.0 mL min^−1^ and a controlled temperature of 25 °C. The injection volume was 10 μL, and the detection wavelength was set at λ = 254 nm. The mobile phase consisted of acetonitrile + 0.1% TFA and water + 0.1% TFA. A gradient method was developed and is detailed in Table 3.

#### 3.3.3. Chiral HPLC Method for Identification of AR-15512 and ent-AR15512, **5**

*Sample concentration*. Solid sample 1 mg AR-15512 in 1 mL 2-propanol; solid sample 1 mg compound **5** in 1 mL 2-propanol; and solid sample mixture 1 mg AR-15512/1 mg compound **5** in 2 mL 2-propanol; then, from the first two solutions, a new solution was prepared with 0.75 mL of AR-15512 solution and 0.25 mL of compound **5** solution.

*Chromatographic conditions*. The employed column was a Chiralpack IC (Daicel Chemical Ind., Ltd., Tokyo, Japan) 5 ODS 4.6 mm × 250 mm (5 μm), with a constant flow of 1.0 mL min^−1^ and a controlled temperature of 25 °C. The injection volume was 10 μL, and the detection wavelength was λ = 254 nm. The mobile phase consisted of isocratic 90% hexane and 10% 2-propanol.

## 4. Conclusions

In this work, two stereoisomers of AR-15512, a TRPM8 agonist currently in clinical trials for the treatment of the very common ophthalmological disorder dry eye, were synthesized and fully characterized. On one hand, its diastereomer at the C-1 was obtained, and its absolute configuration was confirmed by X-ray crystallographic analysis. On the other hand, and to our knowledge, we report for the first time a synthetic sequence to access the enantiomer of AR-15512. In addition, we provide two different HPLC methods designed and developed to identify both stereoisomers and to differentiate them from the clinical candidate AR-15512.

## Figures and Tables

**Table 1:** Retention times of AR-15512 and its diastereomer **4**.

Compound	Retention Time (min)
AR-15512	11.8
*neo*-AR-15512, **4**	15.6

**Table 2:** Retention times of AR-15512 and its enantiomer **5**.

Compound	Retention Time (min)
AR-15512	12.0
*ent*-AR-15512, **5**	10.0

**Table 3:** Detailed gradient for the developed method.

Time (min)	% Acetonitrile + 0.1% TFA	% Water + 0.1% TFA
0	5	95
0.5	5	95
3	65	35
15	65	35
20	95	5
22	95	5
25	5	95

## Data Availability

Not applicable.

## Supplementary Materials

The following are available online, Figure S1. ^1^H, ^13^C, HSQC and COSY NMR and IR spectra of compound **4**, Figure S2. ^1^H, ^13^C, HSQC and COSY NMR and IR spectra of compound **5**, Tables S1–S5. X-ray crystallographic data for compound **4**, Figure S3. HPLC chromatograms of compounds AR-15512 and **4**, Figure S4. HPLC chromatograms of compounds AR-15512 and **5**.

## Sample Availability

Samples of the compounds are available from the authors.
