# Supplementary material for: Synthesis, Characterization and HPLC Analysis of the (1S,2S,5R)-Diastereomer and the Enantiomer of the Clinical Candidate AR-15512
Source: Molecules. 2021 Feb 9;26(4):906. doi: 10.3390/molecules26040906 (PMC7914790; doi:10.3390/molecules26040906)
Supplement: Supplementary file 1 [file molecules-26-00906-s001.pdf]

SUPPLEMENTARY MATERIAL FOR

Synthesis, characterization and HPLC analysis of the (1*S*,2*S*,5*R*)-diastereomer and the enantiomer of the clinical candidate AR-15512

Sergio Rodríguez-Arévalo[a], Eugènia Pujol[a], Sònia Abás[a], Carles Galdeano[b], Carmen Escolano[a] and Santiago Vázquez[a]

[a]*Laboratori de Química Farmacèutica (Unitat Associada al CSIC), Facultat de Farmàcia i Ciències de l'Alimentació, and Institute of Biomedicine (IBUB), Universitat de Barcelona, Av. Joan XXIII 27-31, 08028 Barcelona, Spain*

[b]*Department of Pharmacy and Pharmaceutical Technology and Physical Chemistry, Faculty of Pharmacy and Food Sciences and Institute of Biomedicine, University of Barcelona, Av. Joan XXIII, 27-31, E-08028, Barcelona, Spain*

Table of contents

Figure S1: ^{1}H, ^{13}C, HSQC and COSY, and IR spectra of compound **4**	Page S3
Figure S2: ^{1}H, ^{13}C, HSQC and COSY, and IR spectra of compound **5**	Page S10
Table S1	Page S18
Table S2	Page S19
Table S3	Page S20
Table S4	Page S25
Table S5	Page S26
Figure S3: HPLC chromatograms of AR-15512 and compound **4**	Page S27
Figure S4: HPLC chromatograms of AR-15512 and compound **5**	Page S30

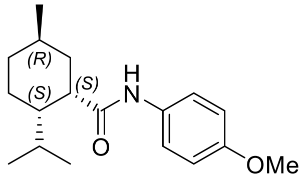

^{1}H (400 MHz, $CDCl_3$)

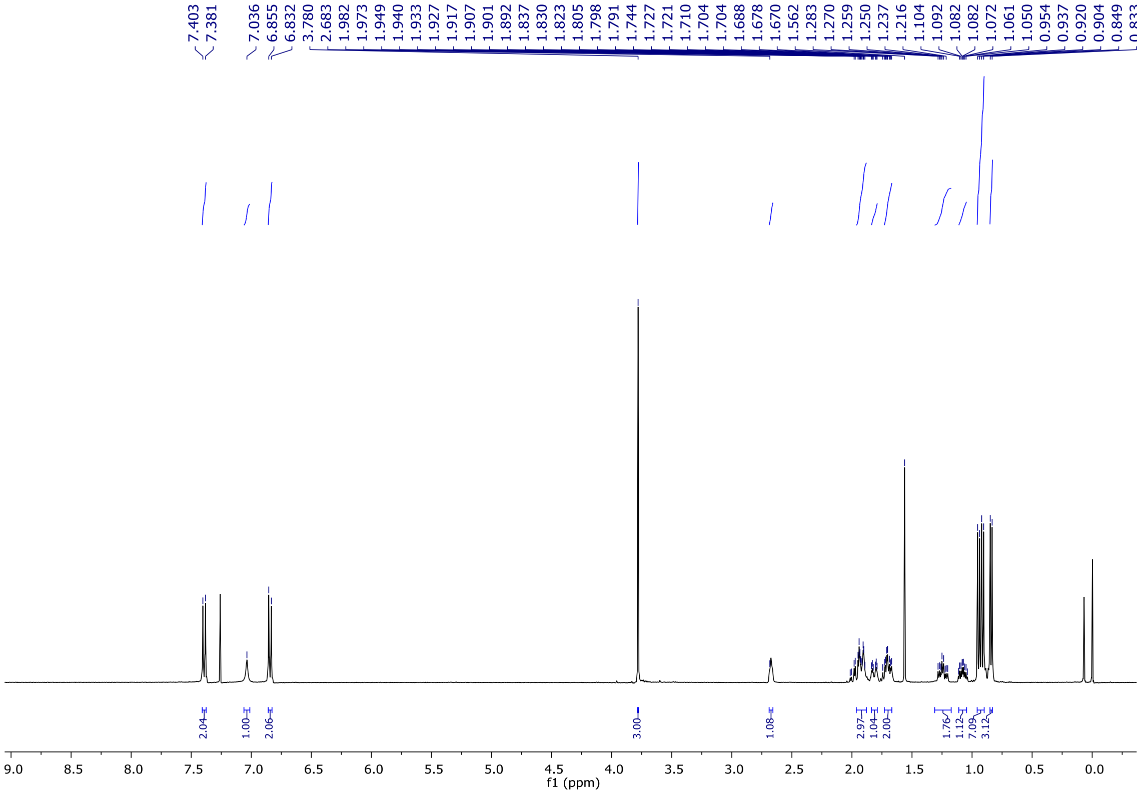

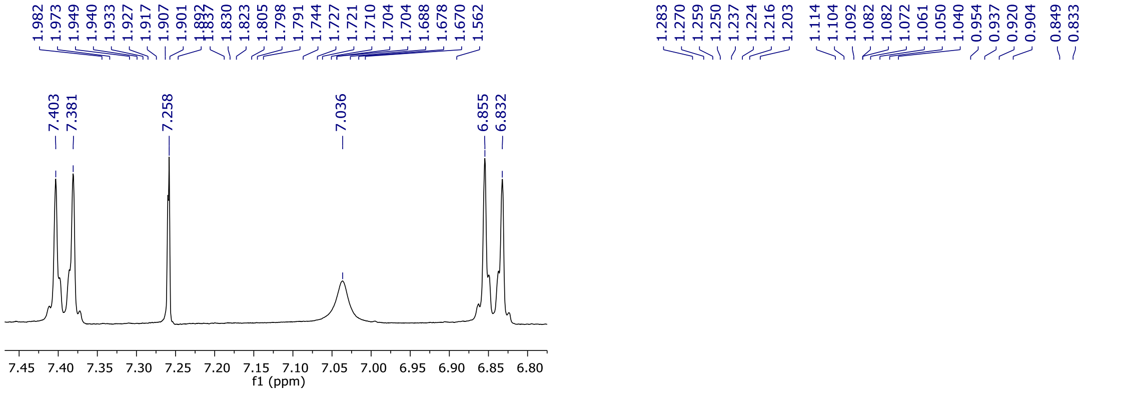

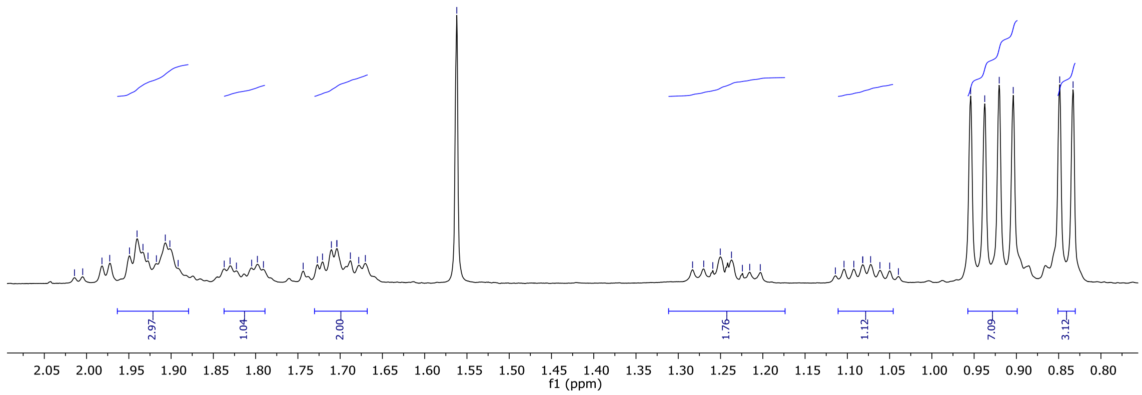

^{13}C (100.6 MHz, $CDCl_3$)

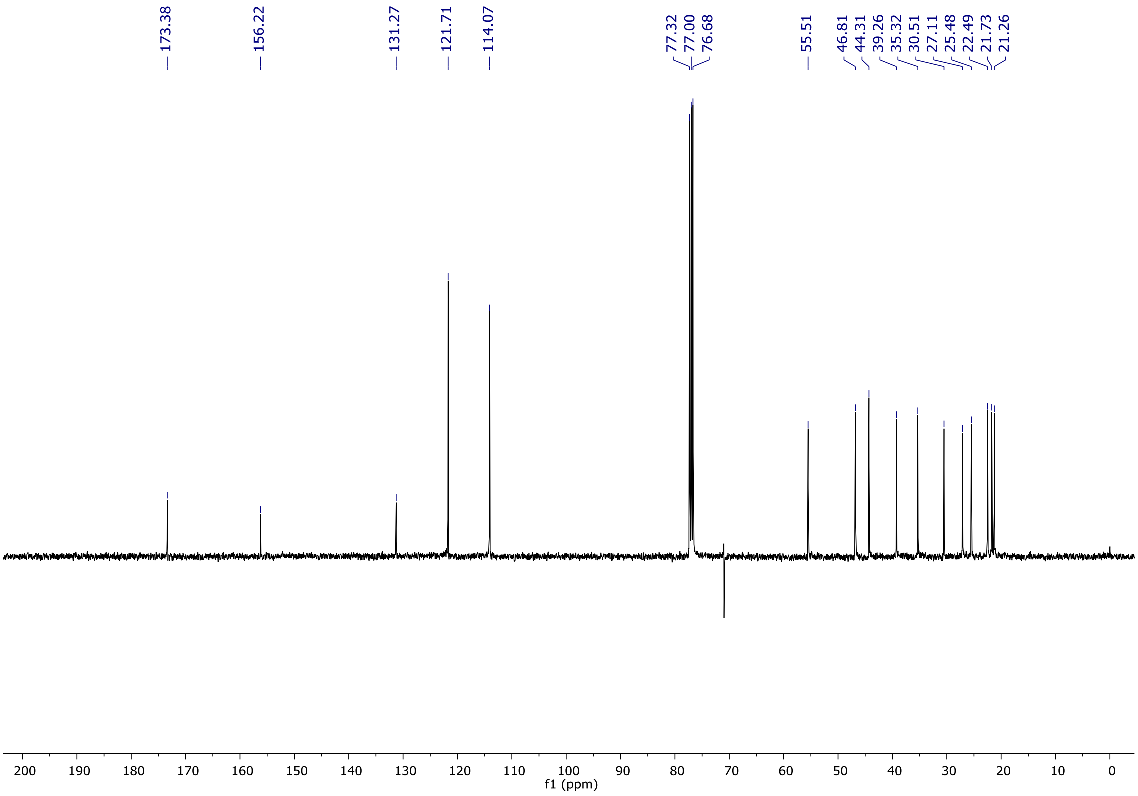

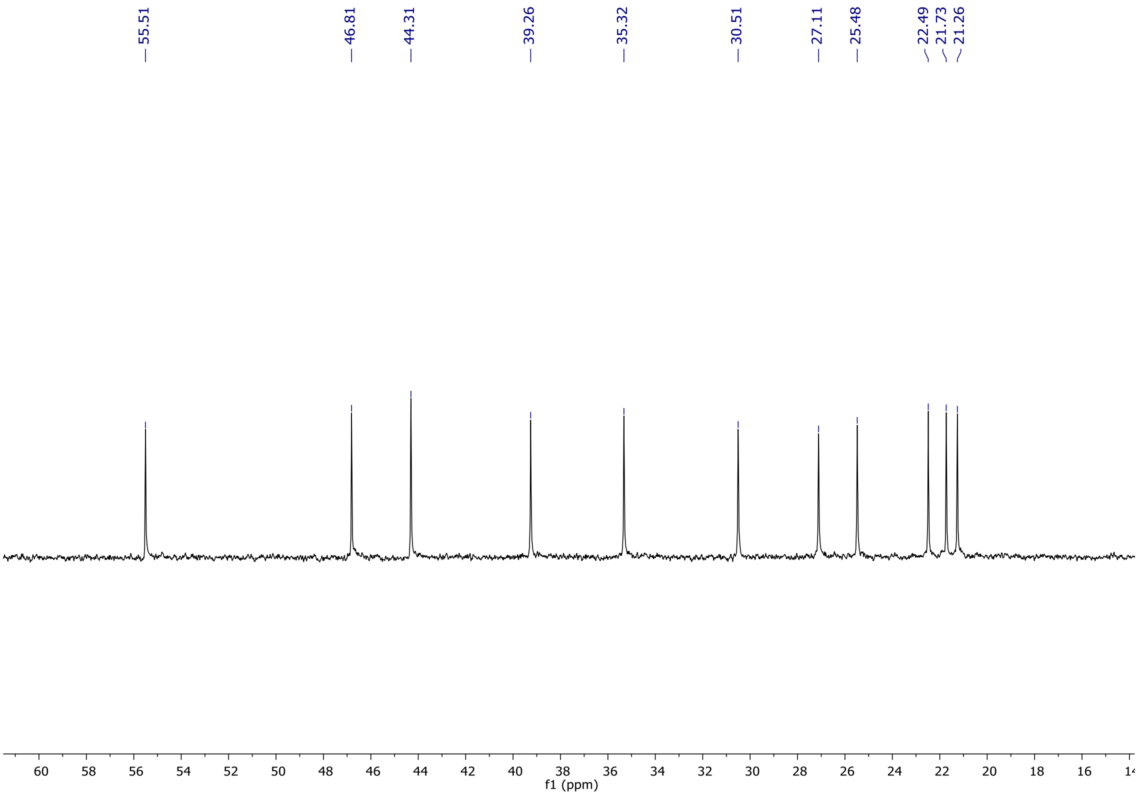

HSQC (400 MHz, CDCl$_3$)

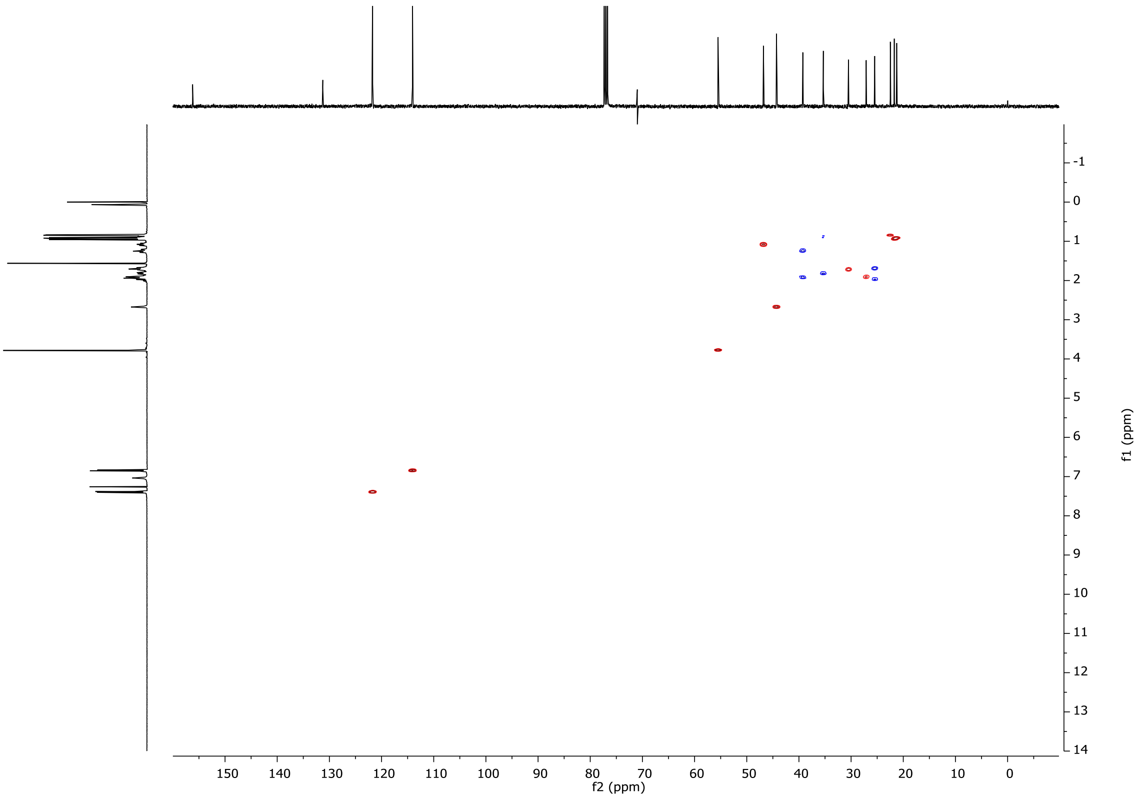

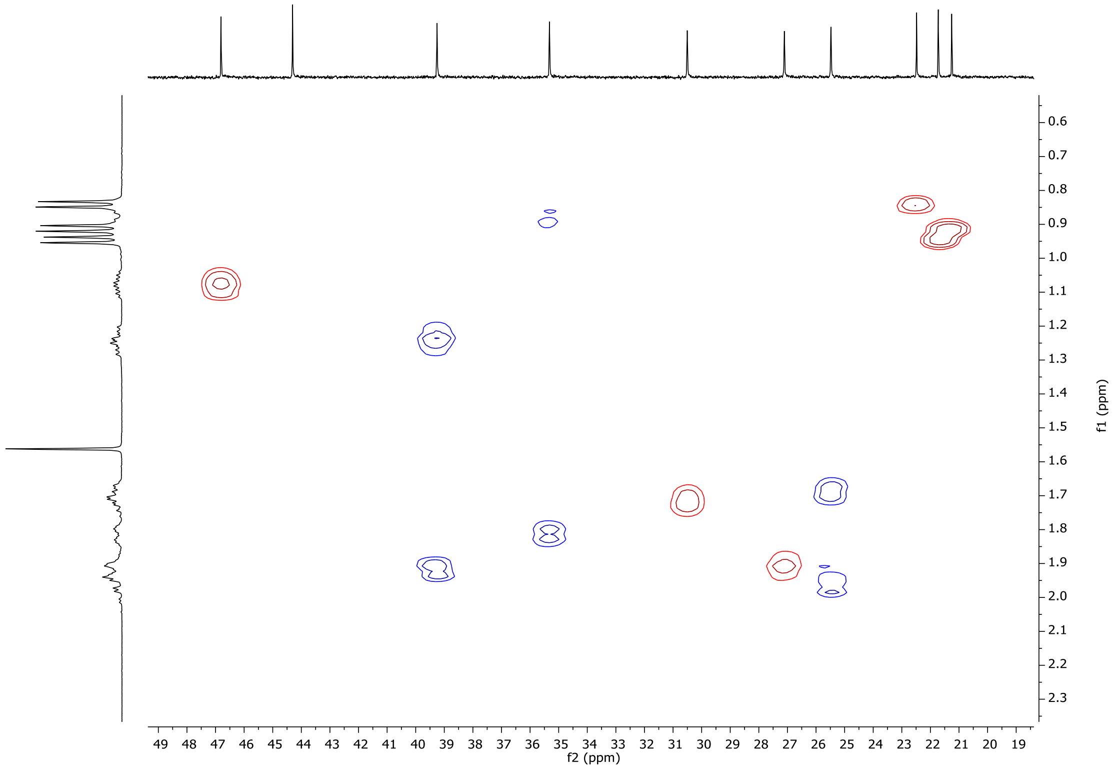

COSY (400 MHz, $CDCl_3$)

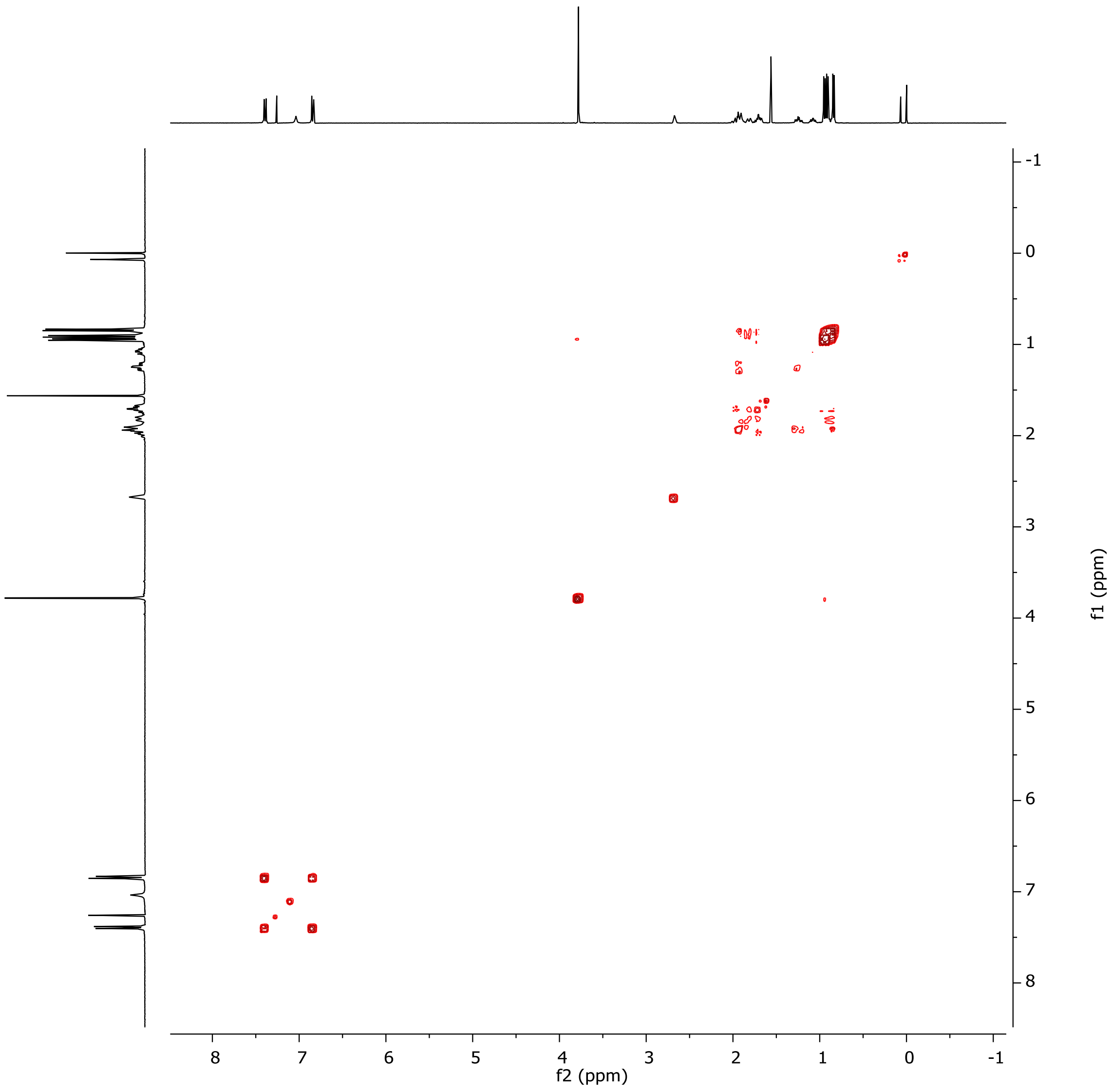

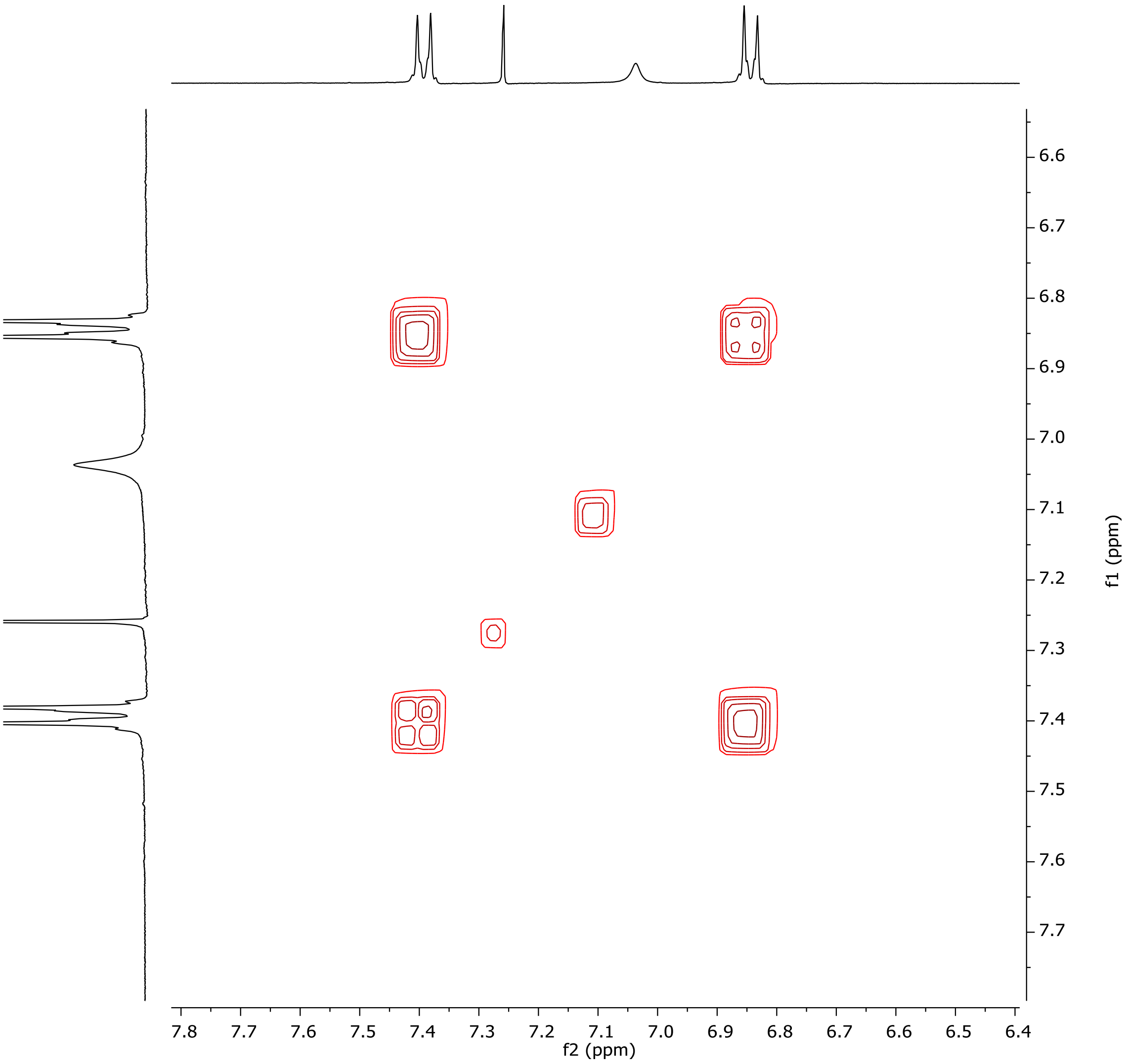

6.6
6.7
6.8
6.9
7.0
7.1
7.2
7.3
7.4
7.5
7.6
7.7
f1 (ppm)
7.8
7.7
7.6
7.5
7.4
7.3
7.2
7.1
7.0
6.9
6.8
6.7
6.6
6.5
6.4
f2 (ppm)

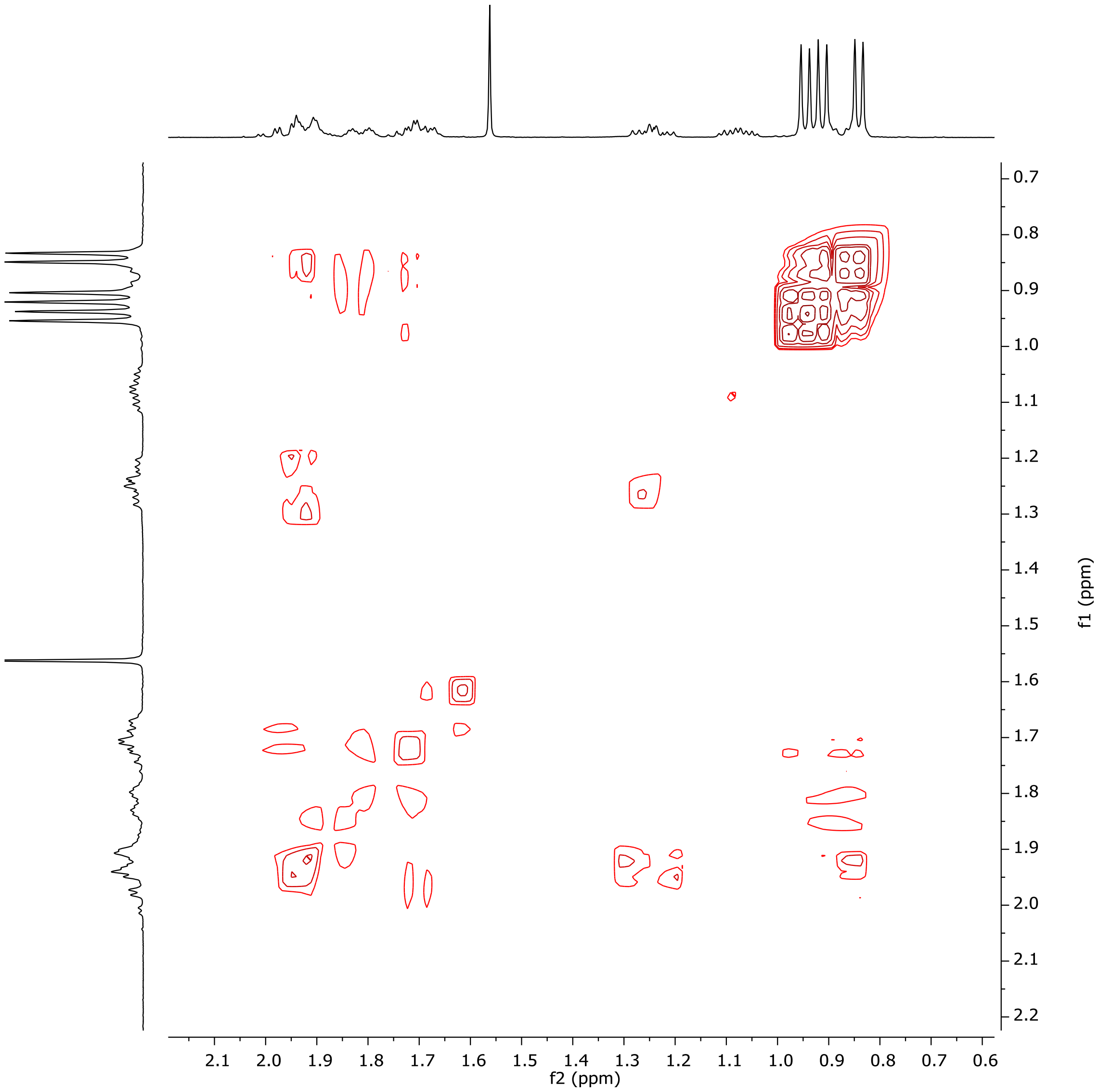
f2 (ppm)
f1 (ppm)

IR spectrum

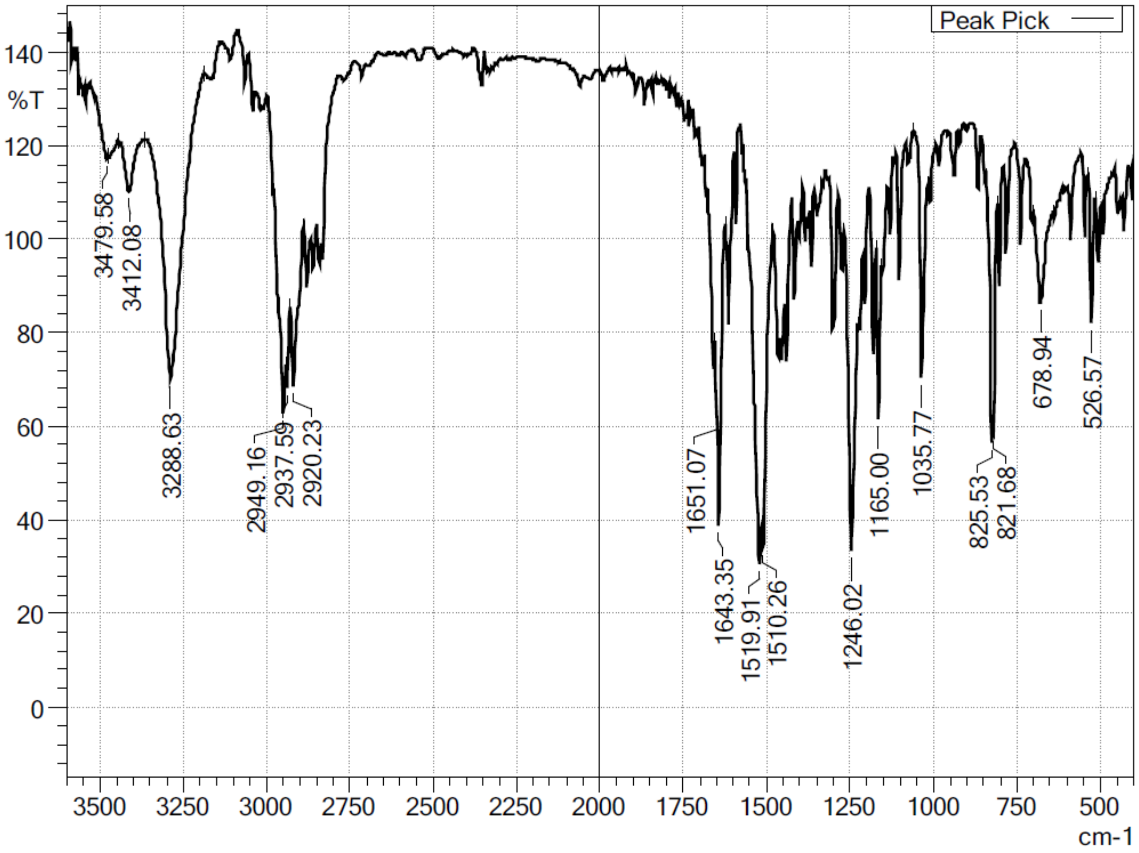

No.	Peak	Intensity	Corr. Intensity	Base (H)	Base (L)	Area	Corr. Area	Area color
1	526.57	82.19	28.92	538.14	514.99	-10.658	246.570	Auto
2	678.94	86.46	19.26	702.09	640.37	124.997	480.292	Auto
3	821.68	58.22	6.93	823.60	808.17	286.005	28.918	Auto
4	825.53	56.33	7.68	844.82	823.60	181.645	-111.872	Auto
5	1035.77	70.27	46.78	1058.92	1016.49	-139.244	606.376	Auto
6	1165.00	61.39	34.97	1172.72	1155.36	323.561	256.670	Auto
7	1246.02	33.26	58.02	1269.16	1224.80	1445.945	1078.110	Auto
8	1510.26	33.97	12.75	1514.12	1481.33	956.741	-7.751	Auto
9	1519.91	30.42	17.46	1554.63	1514.12	1546.766	296.228	Auto
10	1643.35	38.89	33.71	1649.14	1624.06	745.284	327.499	Auto
11	1651.07	62.59	5.80	1654.92	1649.14	184.056	15.567	Auto
12	2920.23	68.49	21.08	2929.87	2889.37	650.521	408.979	Auto
13	2937.59	68.24	8.99	2941.44	2929.87	290.688	51.110	Auto
14	2949.16	62.89	17.99	2978.09	2941.44	658.969	349.963	Auto
15	3288.63	70.06	57.37	3365.78	3186.40	-1256.928	3841.016	Auto
16	3412.08	110.20	10.87	3442.94	3365.78	-1311.649	317.203	Auto
17	3479.58	117.09	1.10	3483.44	3475.73	-136.074	4.329	Auto

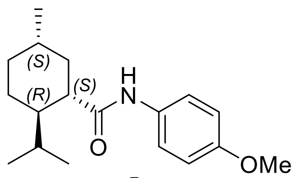

5

^{1}H (400 MHz, $CDCl_3$)

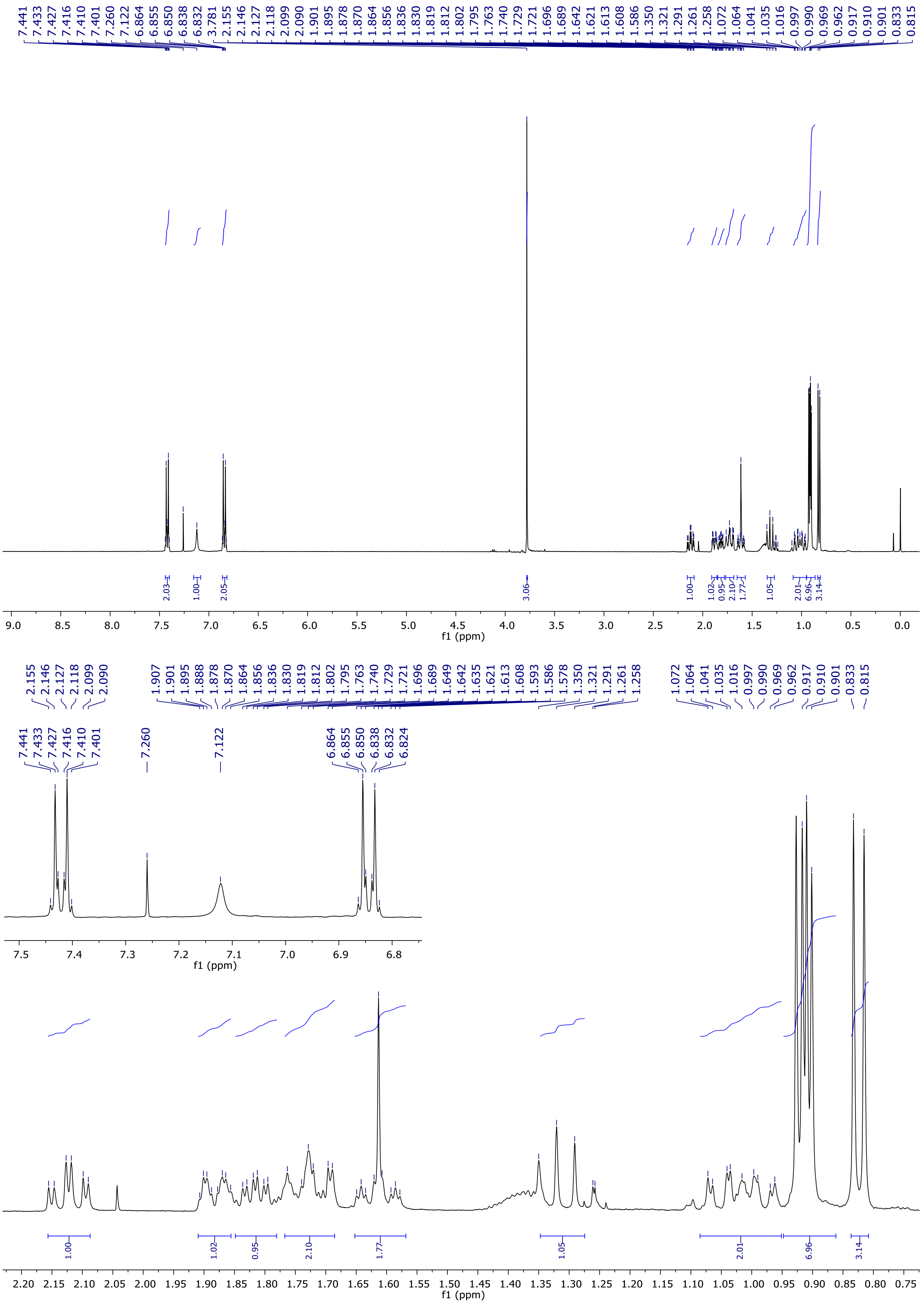

^{13}C (100.6 MHz, $CDCl_3$)

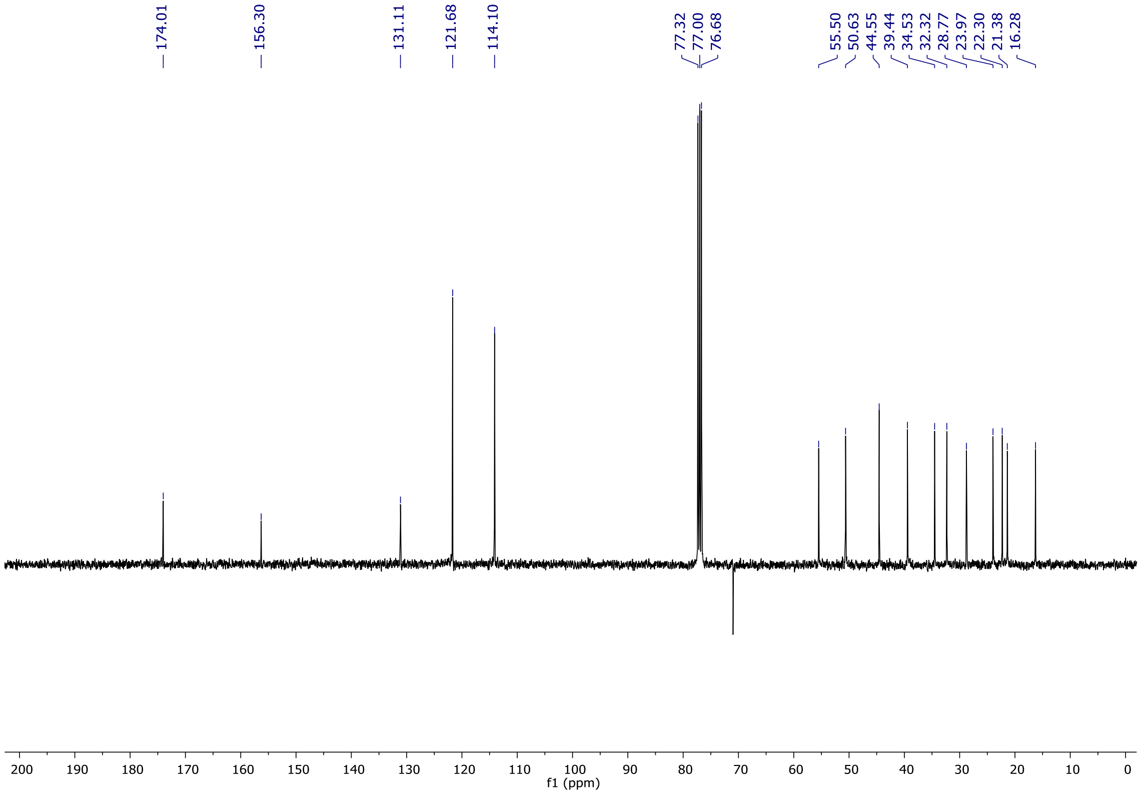

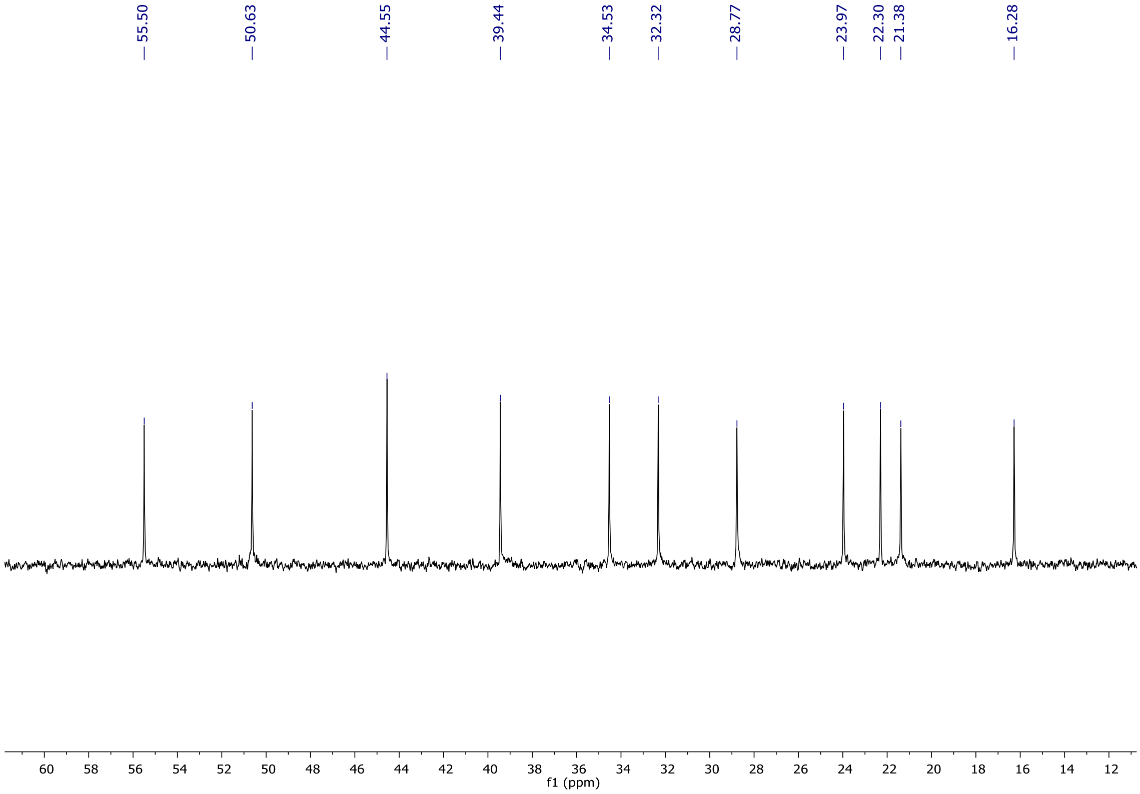

HSQC (400 MHz, $CDCl_3$)

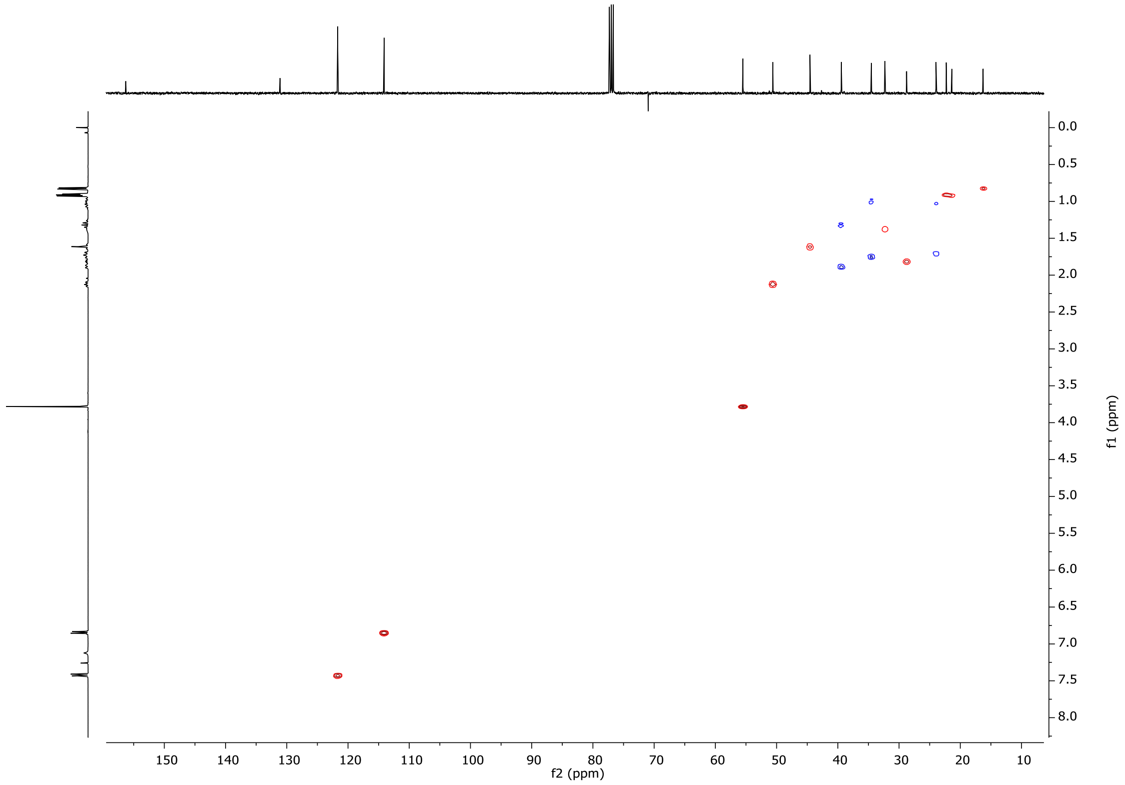

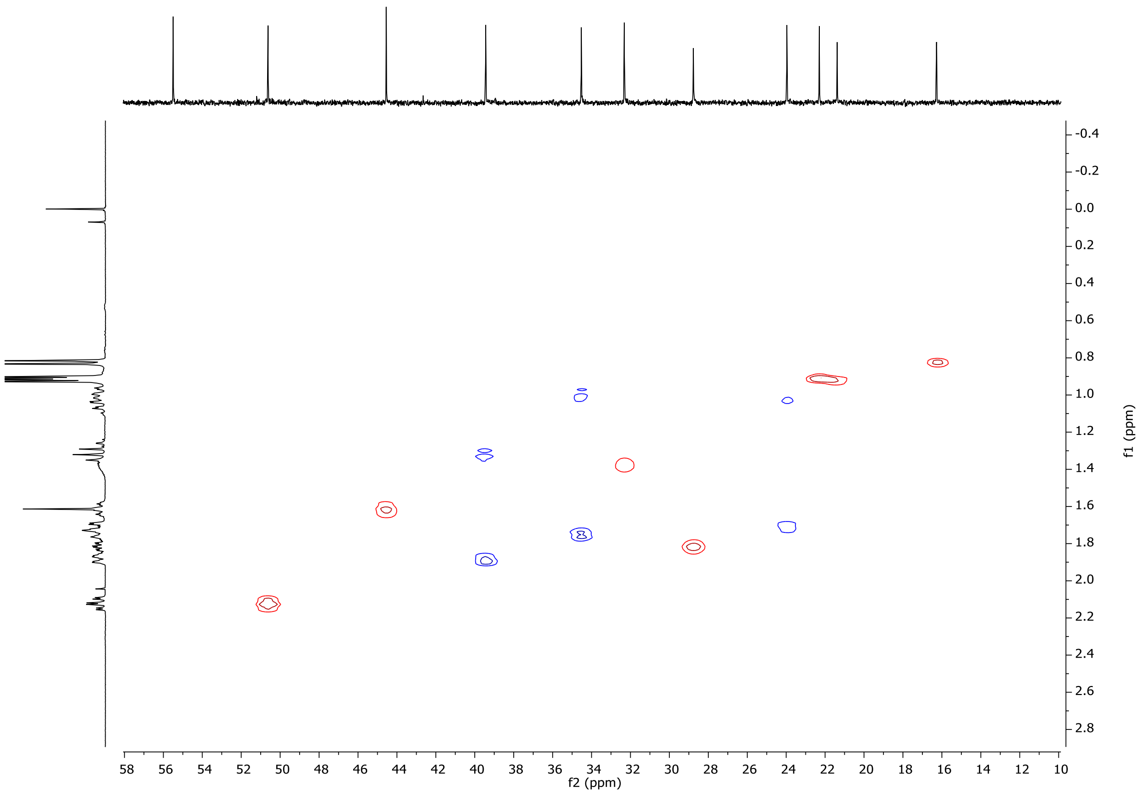

COSY (400 MHz, $CDCl_3$)

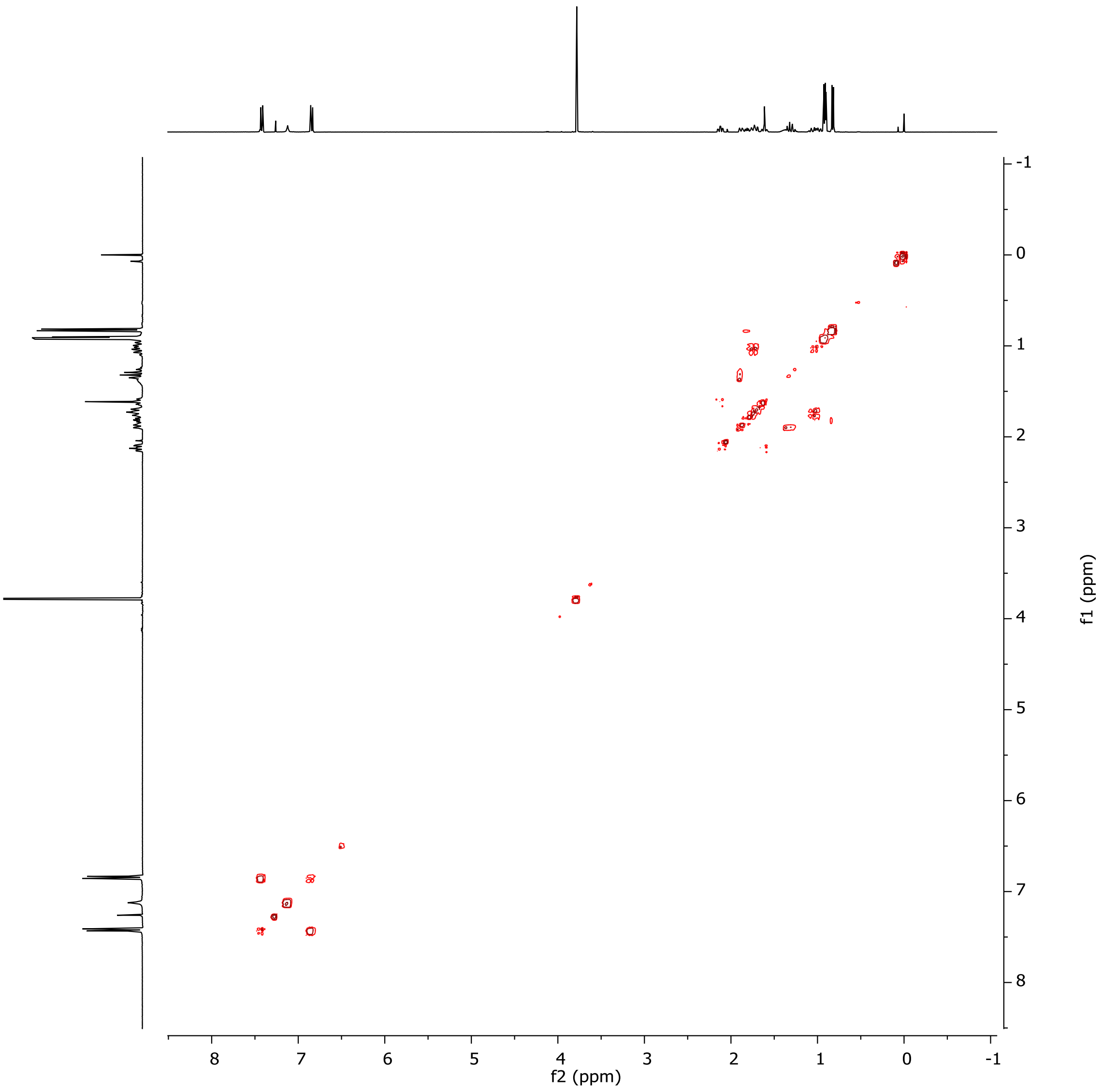

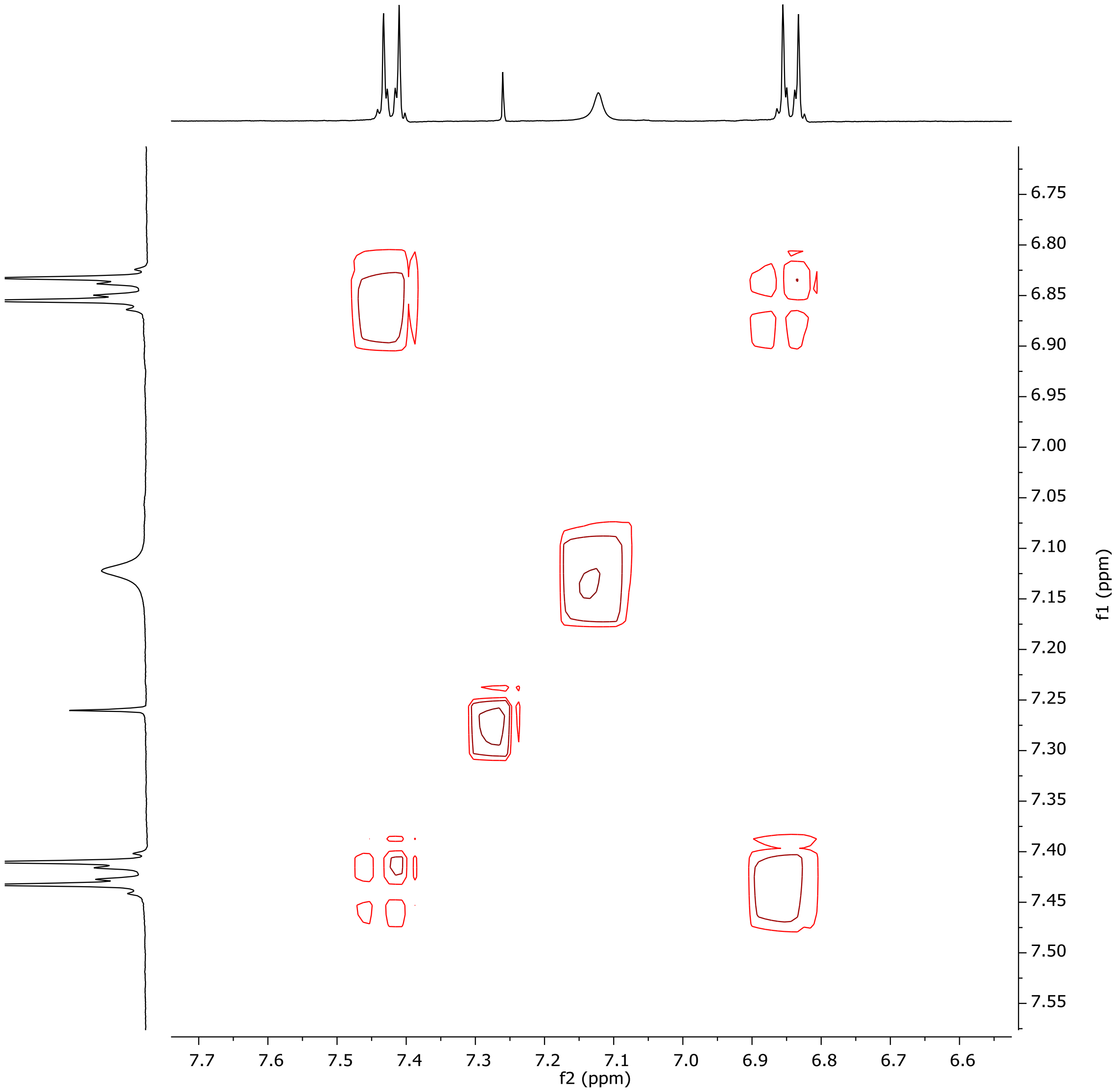
6.75
6.80
6.85
6.90
6.95
7.00
7.05
7.10
7.15
7.20
7.25
7.30
7.35
7.40
7.45
7.50
7.55
f1 (ppm)
7.7
7.6
7.5
7.4
7.3
7.2
7.1
7.0
6.9
6.8
6.7
6.6
f2 (ppm)

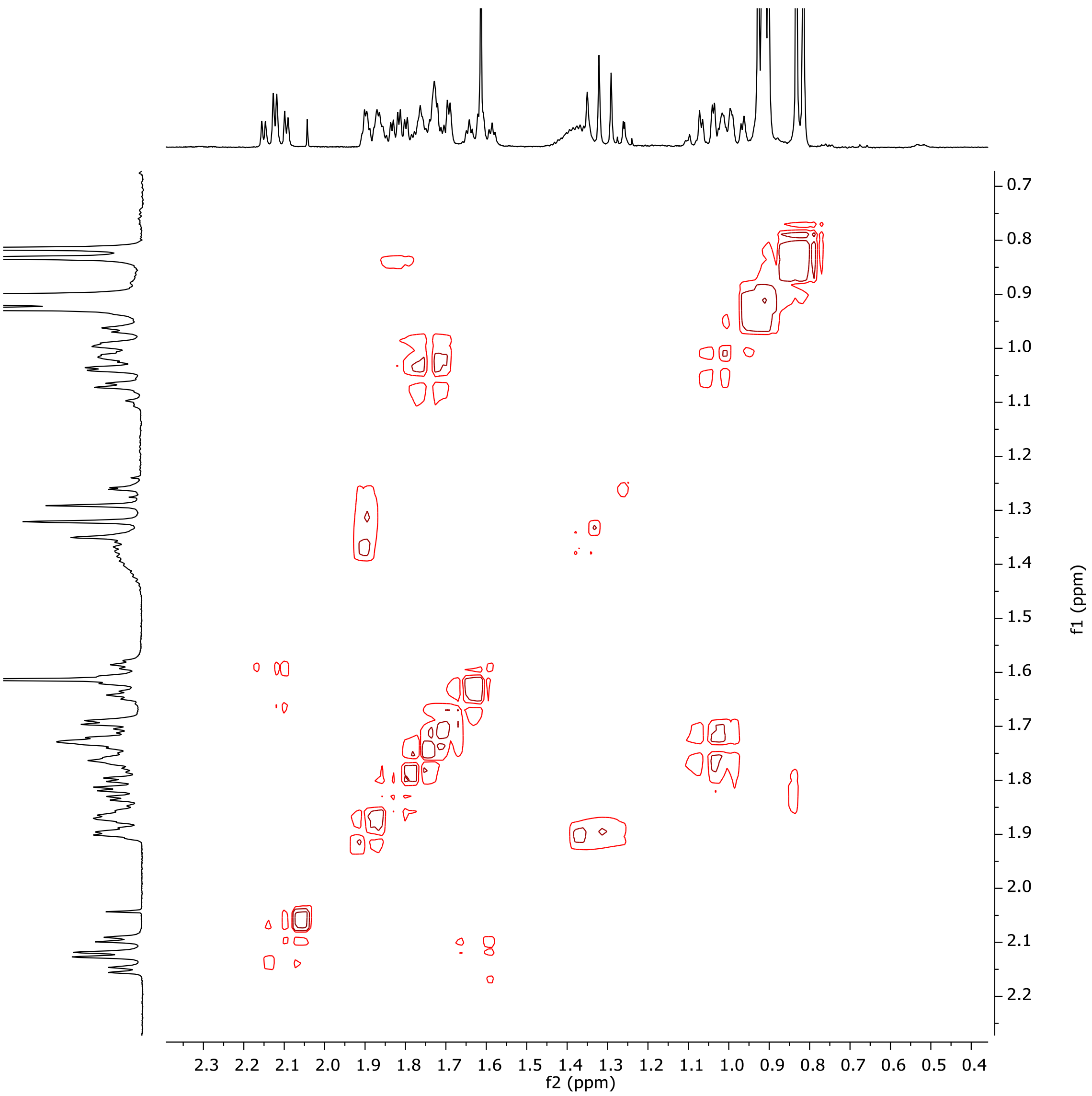
f2 (ppm)
f1 (ppm)

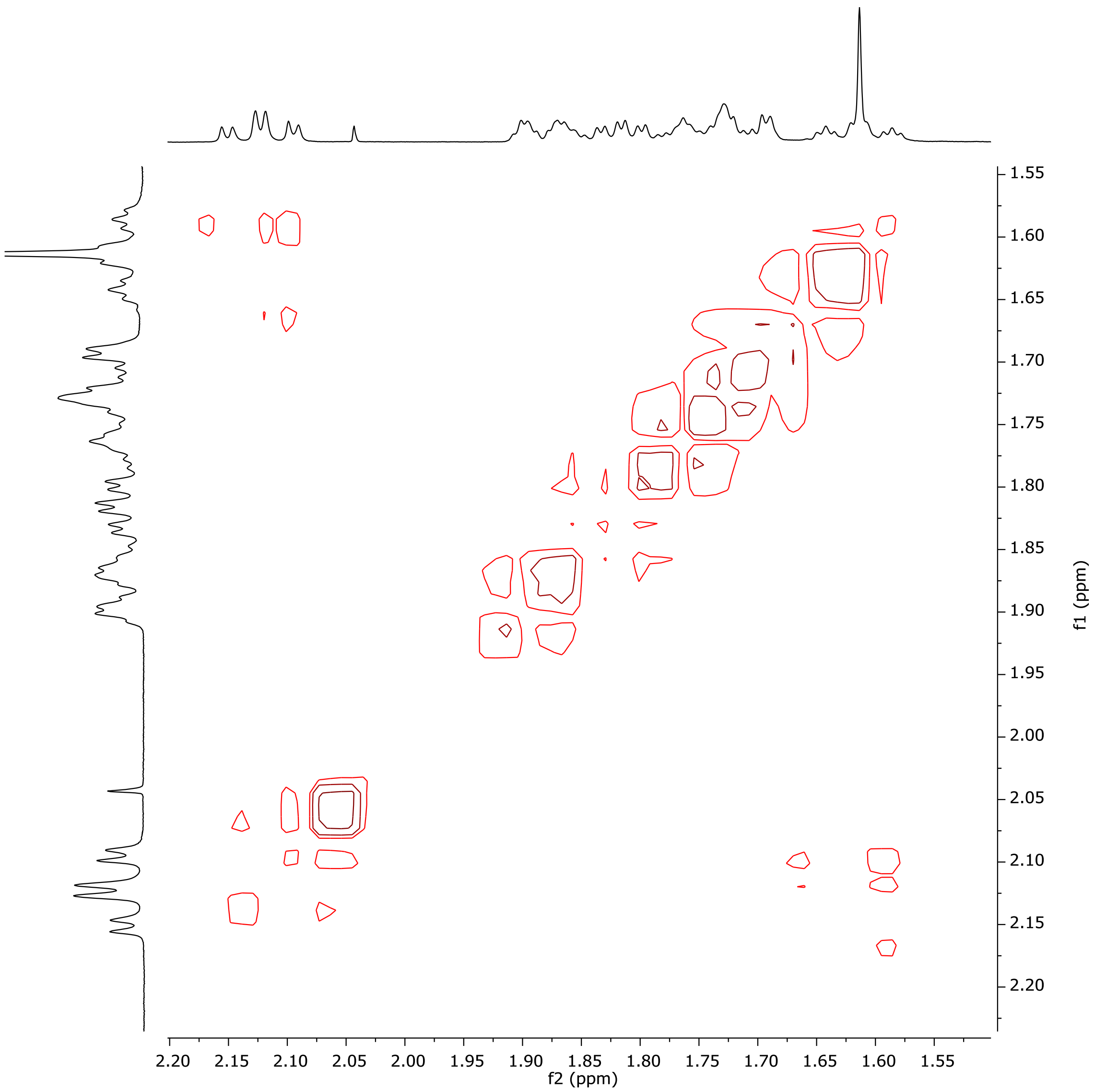

2.20 2.15 2.10 2.05 2.00 1.95 1.90 1.85 1.80 1.75 1.70 1.65 1.60 1.55
f2 (ppm)
1.55 1.60 1.65 1.70 1.75 1.80 1.85 1.90 1.95 2.00 2.05 2.10 2.15 2.20
f1 (ppm)

IR spectrum

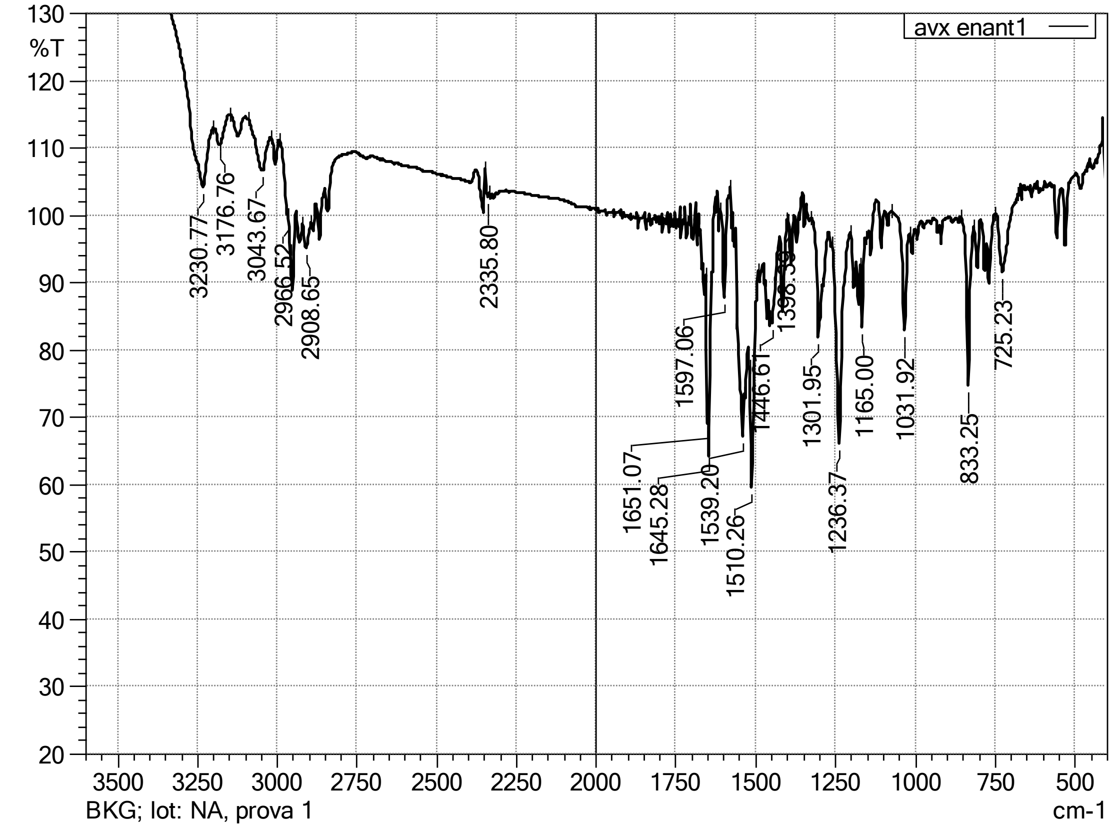

No.	Peak	Intensity	Corr. Intensity	Base (H)	Base (L)	Area	Corr. Area	Area color
1	725.23	91.52	9.66	748.38	677.01	161.357	282.553	Auto
2	833.25	74.61	24.26	856.39	815.89	319.665	278.608	Auto
3	1031.92	82.89	15.13	1074.35	1016.49	252.137	182.864	Auto
4	1165.00	83.30	10.53	1170.79	1153.43	153.352	57.664	Auto
5	1236.37	66.02	30.41	1259.52	1201.65	801.264	603.562	Auto
6	1301.95	81.86	11.01	1325.10	1290.38	286.531	97.526	Auto
7	1398.39	98.84	0.29	1400.32	1396.46	3.900	0.564	Auto
8	1446.61	83.95	3.06	1452.40	1436.97	214.817	25.390	Auto
9	1510.26	59.55	22.06	1517.98	1490.97	662.537	248.654	Auto
10	1539.20	67.14	9.16	1554.63	1533.41	556.720	103.606	Auto
11	1597.06	87.67	14.52	1610.56	1577.77	71.476	152.944	Auto
12	1645.28	64.14	12.70	1649.14	1624.06	350.565	34.900	Auto
13	1651.07	68.93	9.49	1654.92	1649.14	133.541	26.869	Auto
14	2335.80	102.72	1.53	2345.44	2331.94	-46.527	21.211	Auto
15	2908.65	95.17	3.50	2918.30	2891.30	86.666	51.988	Auto
16	2966.52	99.89	0.88	2991.59	2964.59	-169.100	-20.629	Auto
17	3043.67	106.55	5.91	3089.96	3018.60	-717.299	201.286	Auto
18	3176.76	110.37	0.37	3178.69	3145.90	-425.900	-8.787	Auto
19	3230.77	104.17	12.96	3387.00	3197.98	-3922.012	783.082	Auto

Table S1: Crystal data and structure refinement for compound **4**, (1*S*, 2*S*, 5*R*)-diastereoisomer of AR-15512.

Identification code	cu_D29VB58A_0m_a	
Empirical formula	C18 H27 N O2	
Formula weight	289.40	
Temperature	100(2) K	
Wavelength	1.54178 Å	
Crystal system	Orthorhombic	
Space group	P 21 21 21	
Unit cell dimensions	a = 9.1371(2) Å	α= 90°.
	b = 10.3821(3) Å	β= 90°.
	c = 17.4893(4) Å	γ = 90°.
Volume	1659.08(7) $Å^3$	
Z	4	
Density (calculated)	1.159 Mg/m^3	
Absorption coefficient	0.583 mm^{-1}	
F(000)	632	
Crystal size	0.576 x 0.101 x 0.046 mm^3	
Theta range for data collection	5.462 to 59.070°.	
Index ranges	-10<=h<=9, -7<=k<=11, -19<=l<=18	
Reflections collected	8003	
Independent reflections	2370 [R(int) = 0.0219]	
Completeness to theta = 67.679°	79.9 %	
Absorption correction	Semi-empirical from equivalents	
Max. and min. transmission	0.7516 and 0.6145	
Refinement method	Full-matrix least-squares on F^2	
Data / restraints / parameters	2370 / 0 / 194	
Goodness-of-fit on F^2	1.103	
Final R indices [I>2sigma(I)]	R1 = 0.0280, wR2 = 0.0725	
R indices (all data)	R1 = 0.0295, wR2 = 0.0734	
Absolute structure parameter	0.05(7)	
Extinction coefficient	n/a	
Largest diff. peak and hole	0.160 and -0.183 e.$Å^{-3}$	

Table S2: Atomic coordinates (x 10^4) and equivalent isotropic displacement parameters ($Å^2$x 10^3) for cu_D29VB58A_0m_a. U(eq) is defined as one third of the trace of the orthogonalized U^{ij} tensor.

	x	y	z	U(eq)
O(1)	8842(1)	6625(1)	5102(1)	17(1)
O(2)	9061(2)	10230(1)	8006(1)	30(1)
N(1)	6818(2)	7757(2)	5429(1)	17(1)
C(1)	4614(2)	4898(2)	5506(1)	28(1)
C(2)	6154(3)	2931(2)	5385(1)	30(1)
C(3)	6125(2)	4392(2)	5276(1)	19(1)
C(4)	6561(2)	4791(2)	4457(1)	18(1)
C(5)	7966(2)	4141(2)	4182(1)	21(1)
C(6)	8420(2)	4567(2)	3382(1)	23(1)
C(7)	8621(2)	6016(2)	3329(1)	19(1)
C(8)	9097(2)	6434(2)	2534(1)	30(1)
C(9)	7208(2)	6678(2)	3567(1)	19(1)
C(10)	6633(2)	6281(2)	4361(1)	16(1)
C(11)	7542(2)	6896(2)	4989(1)	15(1)
C(12)	7416(2)	8362(2)	6091(1)	16(1)
C(13)	8059(2)	7639(2)	6665(1)	19(1)
C(14)	8612(2)	8221(2)	7316(1)	20(1)
C(15)	8520(2)	9552(2)	7395(1)	20(1)
C(16)	7856(2)	10277(2)	6828(1)	22(1)
C(17)	7306(2)	9687(2)	6180(1)	20(1)
C(18)	9588(3)	9513(2)	8642(1)	34(1)

Table S3: Bond lengths [Å] and angles [°] for cu_D29VB58A_0m_a.

O(1)-C(11)	1.237(2)
O(2)-C(15)	1.372(2)
O(2)-C(18)	1.423(3)
N(1)-C(11)	1.352(2)
N(1)-C(12)	1.426(2)
N(1)-H(1N)	0.8800
C(1)-C(3)	1.530(3)
C(1)-H(1A)	0.9800
C(1)-H(1B)	0.9800
C(1)-H(1C)	0.9800
C(2)-C(3)	1.529(3)
C(2)-H(2A)	0.9800
C(2)-H(2B)	0.9800
C(2)-H(2C)	0.9800
C(3)-C(4)	1.544(3)
C(3)-H(00F)	1.0000
C(4)-C(5)	1.528(3)
C(4)-C(10)	1.557(3)
C(4)-H(4)	1.0000
C(5)-C(6)	1.525(3)
C(5)-H(5A)	0.9900
C(5)-H(5B)	0.9900
C(6)-C(7)	1.518(3)
C(6)-H(6A)	0.9900
C(6)-H(6B)	0.9900
C(7)-C(8)	1.520(3)
C(7)-C(9)	1.521(3)
C(7)-H(7)	1.0000
C(8)-H(8A)	0.9800
C(8)-H(8B)	0.9800
C(8)-H(8C)	0.9800
C(9)-C(10)	1.541(3)

C(9)-H(9A)	0.9900
C(9)-H(9B)	0.9900
C(10)-C(11)	1.517(3)
C(10)-H(10)	1.0000
C(12)-C(13)	1.384(3)
C(12)-C(17)	1.388(3)
C(13)-C(14)	1.385(3)
C(13)-H(13)	0.9500
C(14)-C(15)	1.391(3)
C(14)-H(14)	0.9500
C(15)-C(16)	1.386(3)
C(16)-C(17)	1.383(3)
C(16)-H(16)	0.9500
C(17)-H(17)	0.9500
C(18)-H(18A)	0.9800
C(18)-H(18B)	0.9800
C(18)-H(18C)	0.9800
C(15)-O(2)-C(18)	117.50(16)
C(11)-N(1)-C(12)	124.45(17)
C(11)-N(1)-H(1N)	117.8
C(12)-N(1)-H(1N)	117.8
C(3)-C(1)-H(1A)	109.5
C(3)-C(1)-H(1B)	109.5
H(1A)-C(1)-H(1B)	109.5
C(3)-C(1)-H(1C)	109.5
H(1A)-C(1)-H(1C)	109.5
H(1B)-C(1)-H(1C)	109.5
C(3)-C(2)-H(2A)	109.5
C(3)-C(2)-H(2B)	109.5
H(2A)-C(2)-H(2B)	109.5
C(3)-C(2)-H(2C)	109.5
H(2A)-C(2)-H(2C)	109.5
H(2B)-C(2)-H(2C)	109.5
C(2)-C(3)-C(1)	108.87(18)

C(2)-C(3)-C(4)	112.11(16)
C(1)-C(3)-C(4)	112.64(16)
C(2)-C(3)-H(00F)	107.7
C(1)-C(3)-H(00F)	107.7
C(4)-C(3)-H(00F)	107.7
C(5)-C(4)-C(3)	113.00(16)
C(5)-C(4)-C(10)	111.71(16)
C(3)-C(4)-C(10)	112.14(16)
C(5)-C(4)-H(4)	106.5
C(3)-C(4)-H(4)	106.5
C(10)-C(4)-H(4)	106.5
C(6)-C(5)-C(4)	112.88(16)
C(6)-C(5)-H(5A)	109.0
C(4)-C(5)-H(5A)	109.0
C(6)-C(5)-H(5B)	109.0
C(4)-C(5)-H(5B)	109.0
H(5A)-C(5)-H(5B)	107.8
C(7)-C(6)-C(5)	112.12(16)
C(7)-C(6)-H(6A)	109.2
C(5)-C(6)-H(6A)	109.2
C(7)-C(6)-H(6B)	109.2
C(5)-C(6)-H(6B)	109.2
H(6A)-C(6)-H(6B)	107.9
C(6)-C(7)-C(8)	111.92(18)
C(6)-C(7)-C(9)	109.15(16)
C(8)-C(7)-C(9)	111.37(17)
C(6)-C(7)-H(7)	108.1
C(8)-C(7)-H(7)	108.1
C(9)-C(7)-H(7)	108.1
C(7)-C(8)-H(8A)	109.5
C(7)-C(8)-H(8B)	109.5
H(8A)-C(8)-H(8B)	109.5
C(7)-C(8)-H(8C)	109.5
H(8A)-C(8)-H(8C)	109.5

H(8B)-C(8)-H(8C)	109.5
C(7)-C(9)-C(10)	114.54(16)
C(7)-C(9)-H(9A)	108.6
C(10)-C(9)-H(9A)	108.6
C(7)-C(9)-H(9B)	108.6
C(10)-C(9)-H(9B)	108.6
H(9A)-C(9)-H(9B)	107.6
C(11)-C(10)-C(9)	110.67(15)
C(11)-C(10)-C(4)	111.28(15)
C(9)-C(10)-C(4)	112.15(16)
C(11)-C(10)-H(10)	107.5
C(9)-C(10)-H(10)	107.5
C(4)-C(10)-H(10)	107.5
O(1)-C(11)-N(1)	121.91(17)
O(1)-C(11)-C(10)	123.09(17)
N(1)-C(11)-C(10)	114.99(17)
C(13)-C(12)-C(17)	119.11(18)
C(13)-C(12)-N(1)	120.87(17)
C(17)-C(12)-N(1)	119.98(17)
C(12)-C(13)-C(14)	121.01(18)
C(12)-C(13)-H(13)	119.5
C(14)-C(13)-H(13)	119.5
C(13)-C(14)-C(15)	119.54(19)
C(13)-C(14)-H(14)	120.2
C(15)-C(14)-H(14)	120.2
O(2)-C(15)-C(16)	115.89(17)
O(2)-C(15)-C(14)	124.46(18)
C(16)-C(15)-C(14)	119.65(18)
C(17)-C(16)-C(15)	120.38(18)
C(17)-C(16)-H(16)	119.8
C(15)-C(16)-H(16)	119.8
C(16)-C(17)-C(12)	120.29(19)
C(16)-C(17)-H(17)	119.9
C(12)-C(17)-H(17)	119.9

O(2)-C(18)-H(18A)	109.5
O(2)-C(18)-H(18B)	109.5
H(18A)-C(18)-H(18B)	109.5
O(2)-C(18)-H(18C)	109.5
H(18A)-C(18)-H(18C)	109.5
H(18B)-C(18)-H(18C)	109.5

Symmetry transformations used to generate equivalent atoms:

Table S4: Anisotropic displacement parameters ($Å^2$x 10^3) for cu_D29VB58A_0m_a. The anisotropic displacement factor exponent takes the form: $-2\pi^2[h^2 a^{*2}U^{11} + ... + 2 h k a^* b^* U^{12}]$.

	U^{11}	U^{22}	U^{33}	U^{23}	U^{13}	U^{12}
O(1)	13(1)	19(1)	18(1)	0(1)	-1(1)	0(1)
O(2)	39(1)	26(1)	24(1)	-9(1)	-6(1)	-5(1)
N(1)	13(1)	19(1)	19(1)	-2(1)	0(1)	3(1)
C(1)	25(1)	35(1)	22(1)	6(1)	4(1)	-1(1)
C(2)	38(1)	24(1)	27(1)	3(1)	4(1)	-7(1)
C(3)	19(1)	21(1)	18(1)	1(1)	-1(1)	-3(1)
C(4)	18(1)	18(1)	17(1)	-2(1)	-3(1)	-3(1)
C(5)	23(1)	16(1)	24(1)	-2(1)	0(1)	1(1)
C(6)	22(1)	25(1)	22(1)	-7(1)	2(1)	2(1)
C(7)	16(1)	26(1)	15(1)	1(1)	-2(1)	-2(1)
C(8)	28(1)	42(1)	20(1)	3(1)	4(1)	2(1)
C(9)	18(1)	21(1)	17(1)	2(1)	-3(1)	0(1)
C(10)	12(1)	18(1)	18(1)	1(1)	-1(1)	2(1)
C(11)	16(1)	12(1)	15(1)	5(1)	2(1)	-1(1)
C(12)	13(1)	19(1)	17(1)	-1(1)	4(1)	-1(1)
C(13)	19(1)	15(1)	21(1)	-2(1)	3(1)	-2(1)
C(14)	20(1)	22(1)	20(1)	1(1)	0(1)	-1(1)
C(15)	19(1)	23(1)	19(1)	-4(1)	4(1)	-5(1)
C(16)	25(1)	15(1)	26(1)	-4(1)	5(1)	-1(1)
C(17)	18(1)	20(1)	21(1)	1(1)	2(1)	1(1)
C(18)	40(1)	39(1)	24(1)	-6(1)	-7(1)	-7(1)

Table S5: Hydrogen coordinates (x 10^4) and isotropic displacement parameters (Å^2 x 10^3) for cu_D29VB58A_0m_a.

	x	y	z	U(eq)
H(1N)	5917	7958	5298	20
H(1A)	4596	5838	5457	41
H(1B)	4410	4657	6038	41
H(1C)	3869	4521	5171	41
H(2A)	5539	2522	4995	45
H(2B)	5781	2716	5894	45
H(2C)	7162	2620	5335	45
H(00F)	6858	4774	5635	23
H(4)	5761	4483	4113	21
H(5A)	8766	4341	4544	25
H(5B)	7822	3195	4183	25
H(6A)	9348	4136	3242	27
H(6B)	7663	4294	3010	27
H(7)	9404	6272	3698	23
H(8A)	8351	6178	2161	45
H(8B)	10029	6020	2405	45
H(8C)	9216	7371	2523	45
H(9A)	6445	6485	3181	23
H(9B)	7369	7621	3564	23
H(10)	5613	6619	4410	19
H(13)	8121	6730	6611	22
H(14)	9052	7715	7706	25
H(16)	7778	11184	6884	27
H(17)	6850	10191	5794	23
H(18A)	8808	8952	8835	51
H(18B)	9896	10106	9047	51
H(18C)	10424	8985	8482	51

HPLC chromatograms of AR-15512 and compound 4

SAMPLE INFORMATION

Sample Name:	AR-15512
Sample Type:	Unknown
Vial:	1
Injection #:	8
Injection Volume:	10.00 ul
Run Time:	30.00 Minutes

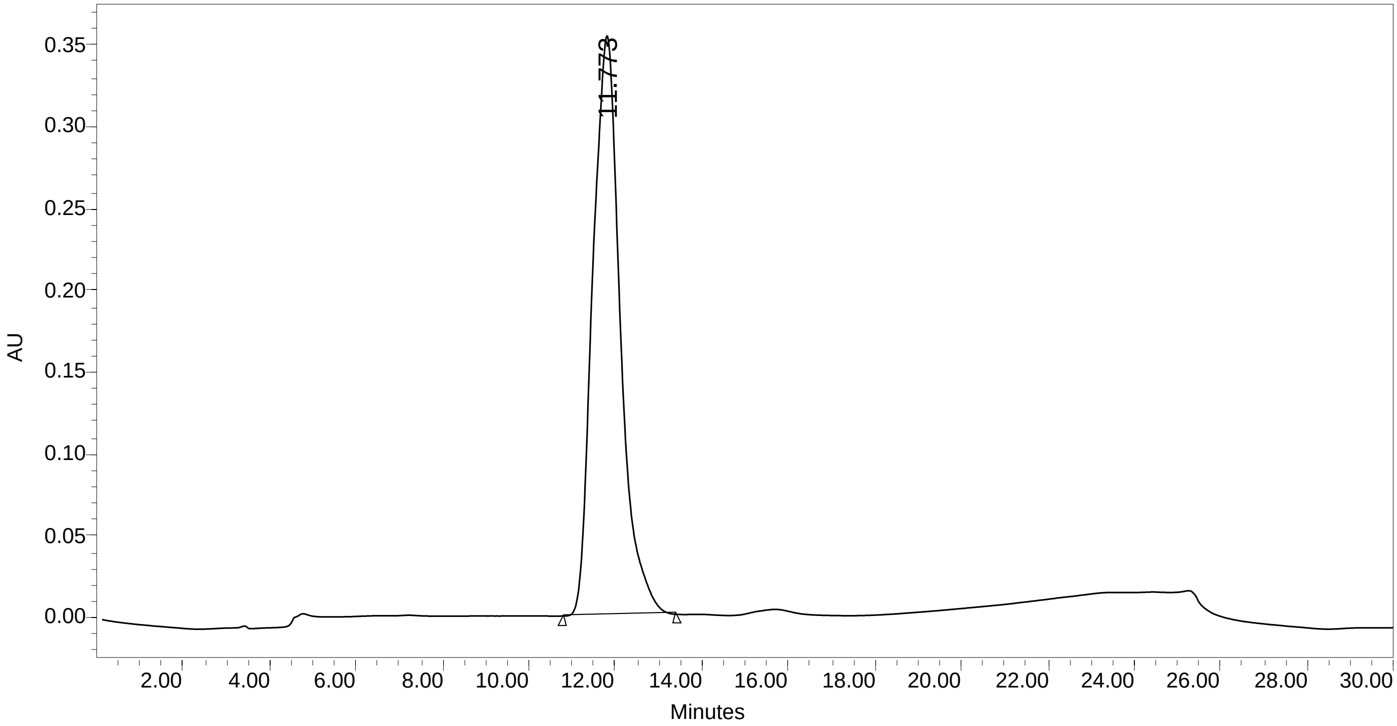

	RT (min)	Area (µV*sec)	% Area	Height (µV)	% Height
1	11.773	15747289	100.00	353932	100.00

S A M P L E   I N F O R M A T I O N

Sample Name:	diastereomer, compound 4
Sample Type:	Unknown
Vial:	1
Injection #:	3
Injection Volume:	10.00 ul
Run Time:	35.00 Minutes

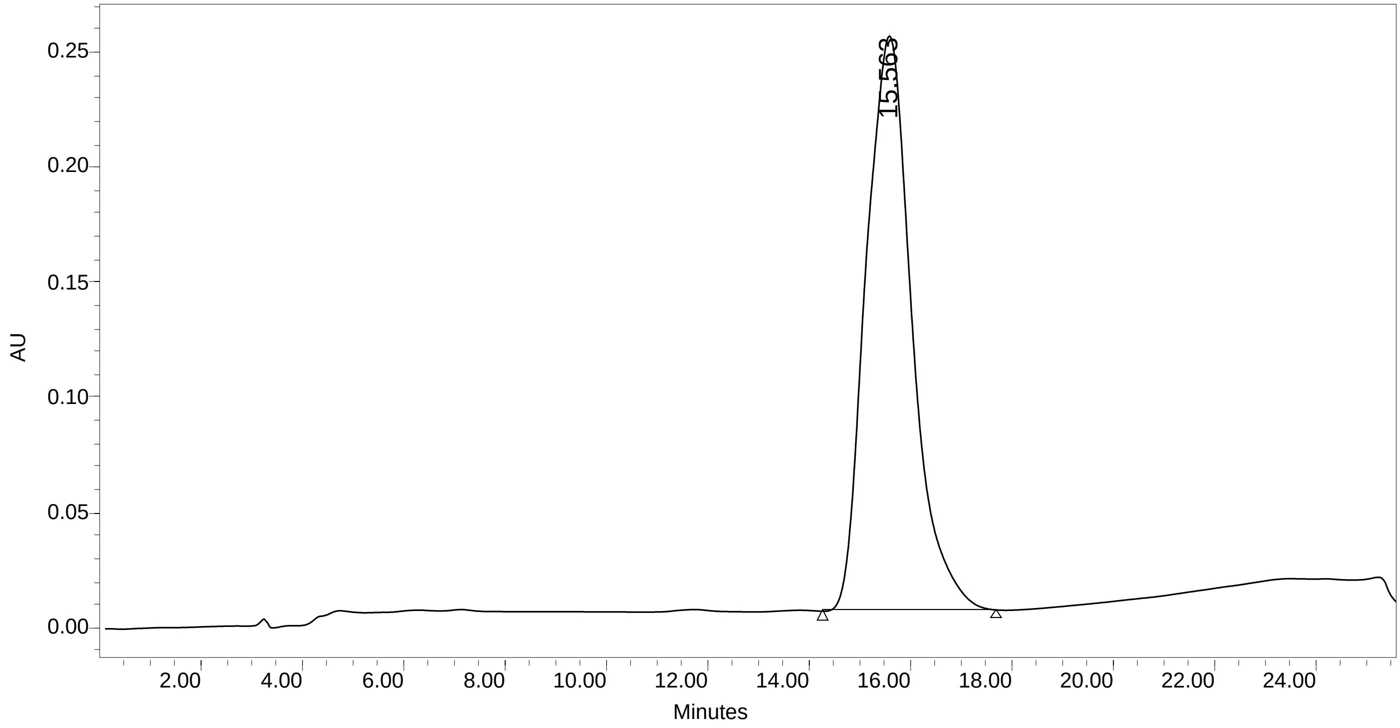

	RT (min)	Area (µV*sec)	% Area	Height (µV)	% Height
1	15.563	15768984	100.00	249674	100.00

SAMPLE INFORMATION

Sample Name:	mixture of AR-15512 and compound 4
Sample Type:	Unknown
Vial:	1
Injection #:	1
Injection Volume:	6.00 ul
Run Time:	35.00 Minutes

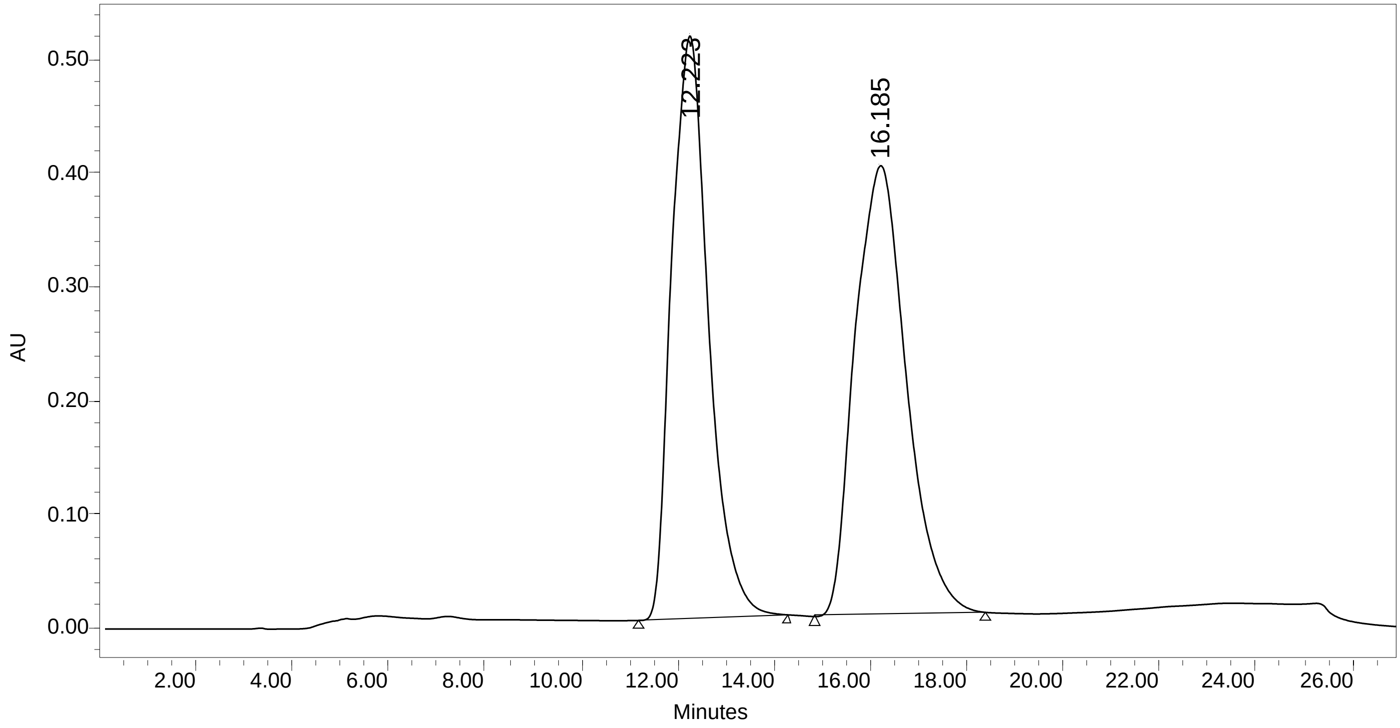

	RT (min)	Area (µV*sec)	% Area	Height (µV)	% Height
1	12.223	27320387	48.25	512990	56.46
2	16.185	29299443	51.75	395629	43.54

HPLC chromatograms of AR-15512 and its enantiomer, compound 5

SAMPLE INFORMATION

Sample Name:	AR-15512 quiral
Sample Type:	Unknown
Vial:	1
Injection #:	4
Injection Volume:	10.00 ul
Run Time:	30.00 Minutes

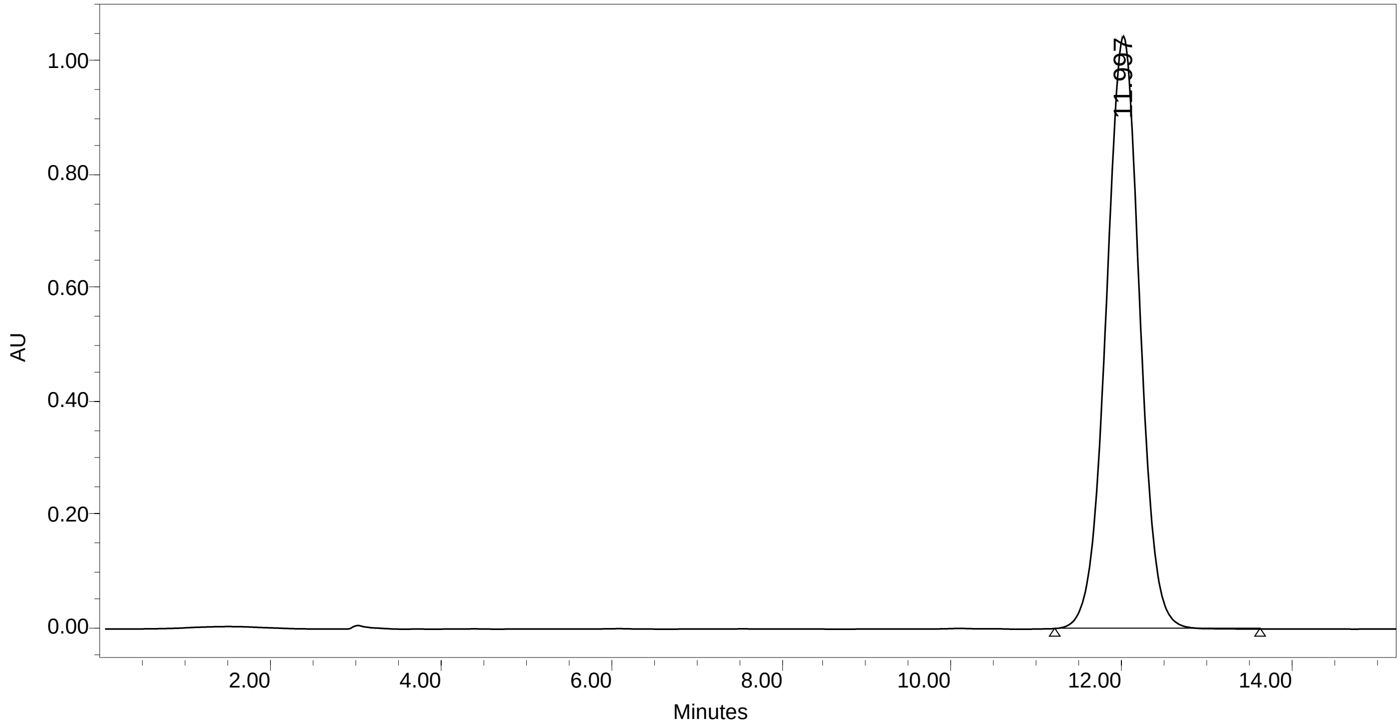

	RT (min)	Area (µV*sec)	% Area	Height (µV)	% Height
1	11.997	28732349	100.00	1046759	100.00

SAMPLE INFORMATION

Sample Name:	enantiomer, compound 5
Sample Type:	Unknown
Vial:	1
Injection #:	2
Injection Volume:	10.00 ul
Run Time:	30.00 Minutes

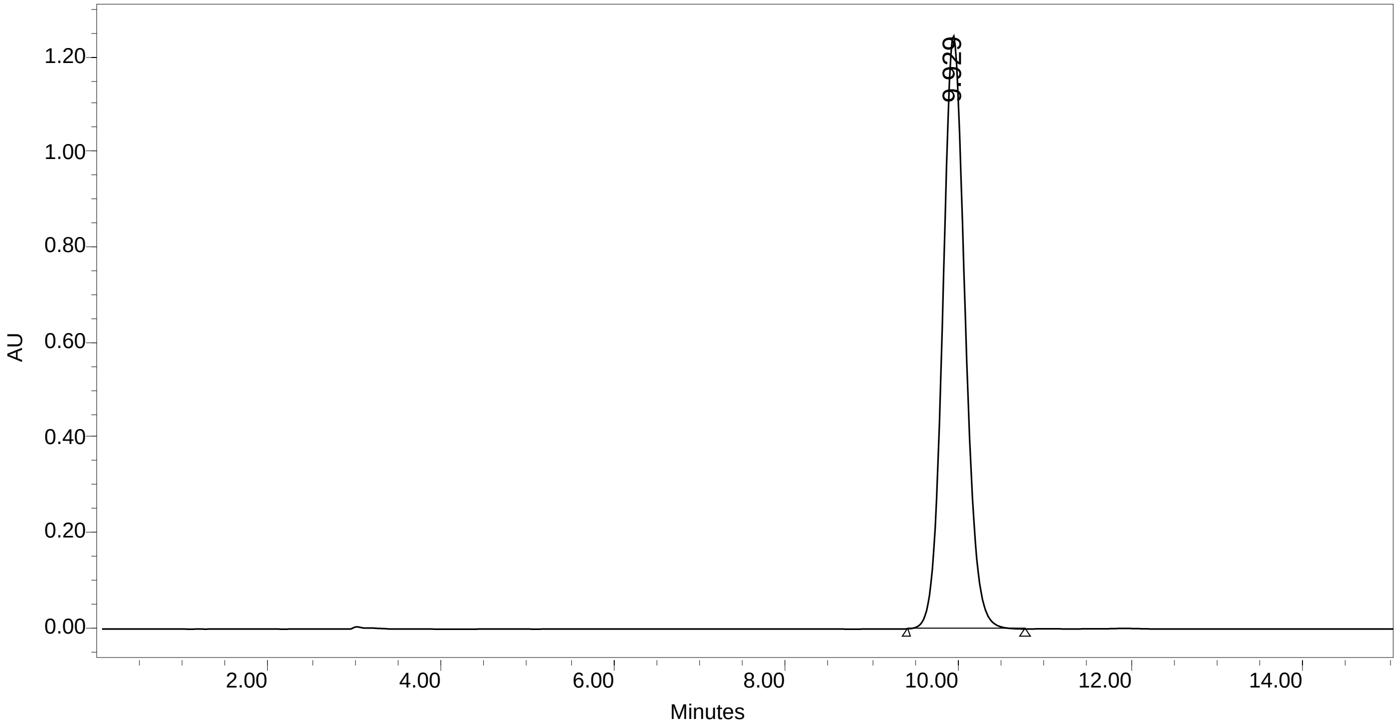

	RT (min)	Area (µV*sec)	% Area	Height (µV)	% Height
1	9.929	22558988	100.00	1245766	100.00

SAMPLE INFORMATION

Sample Name:	Mixture AR-15512 and compound 5
Sample Type:	Unknown
Vial:	1
Injection #:	16
Injection Volume:	10.00 ul
Run Time:	30.00 Minutes

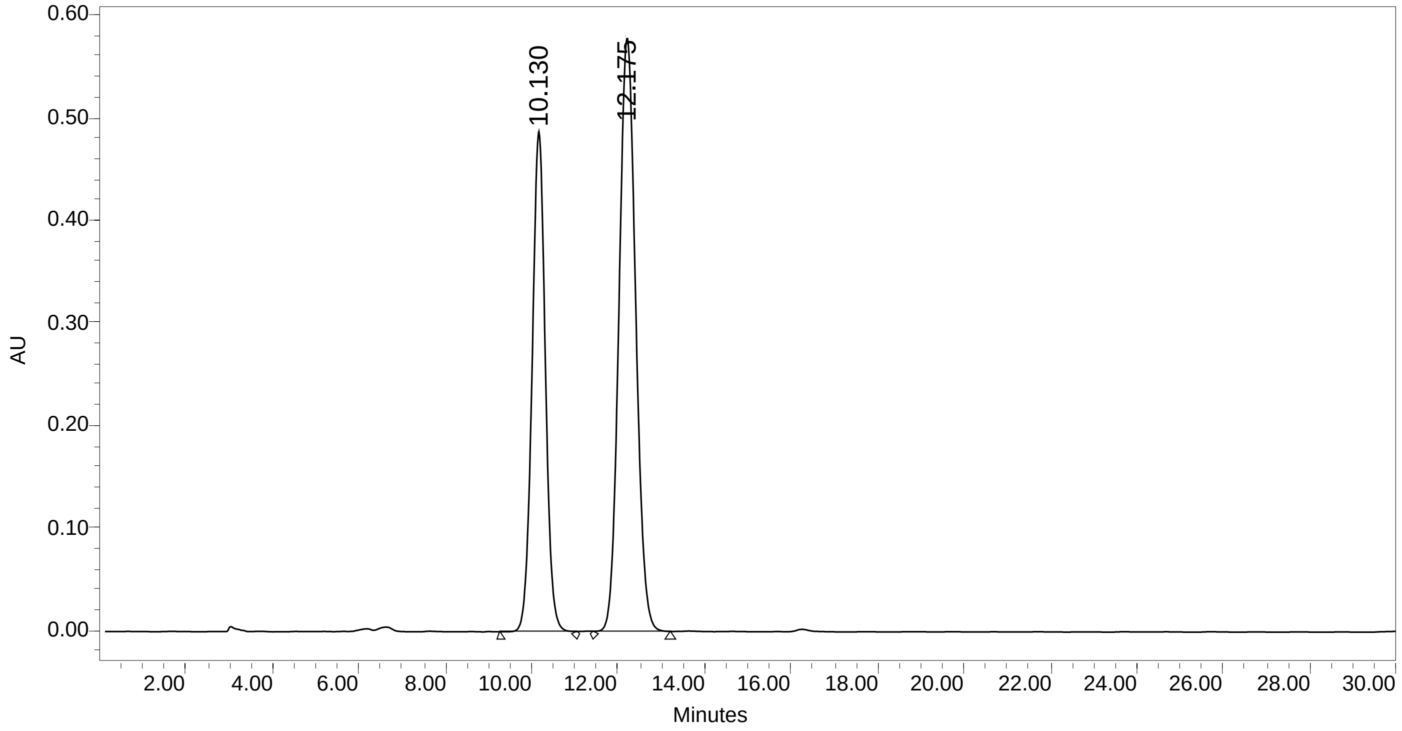

	RT (min)	Area (µV*sec)	% Area	Height (µV)	% Height
1	10.130	10404747	40.00	487679	45.73
2	12.175	15608331	60.00	578793	54.27

SAMPLE INFORMATION

Sample Name:	mixture of enantiomers
Sample Type:	Unknown
Vial:	1
Injection #:	2
Injection Volume:	10.00 ul
Run Time:	30.00 Minutes

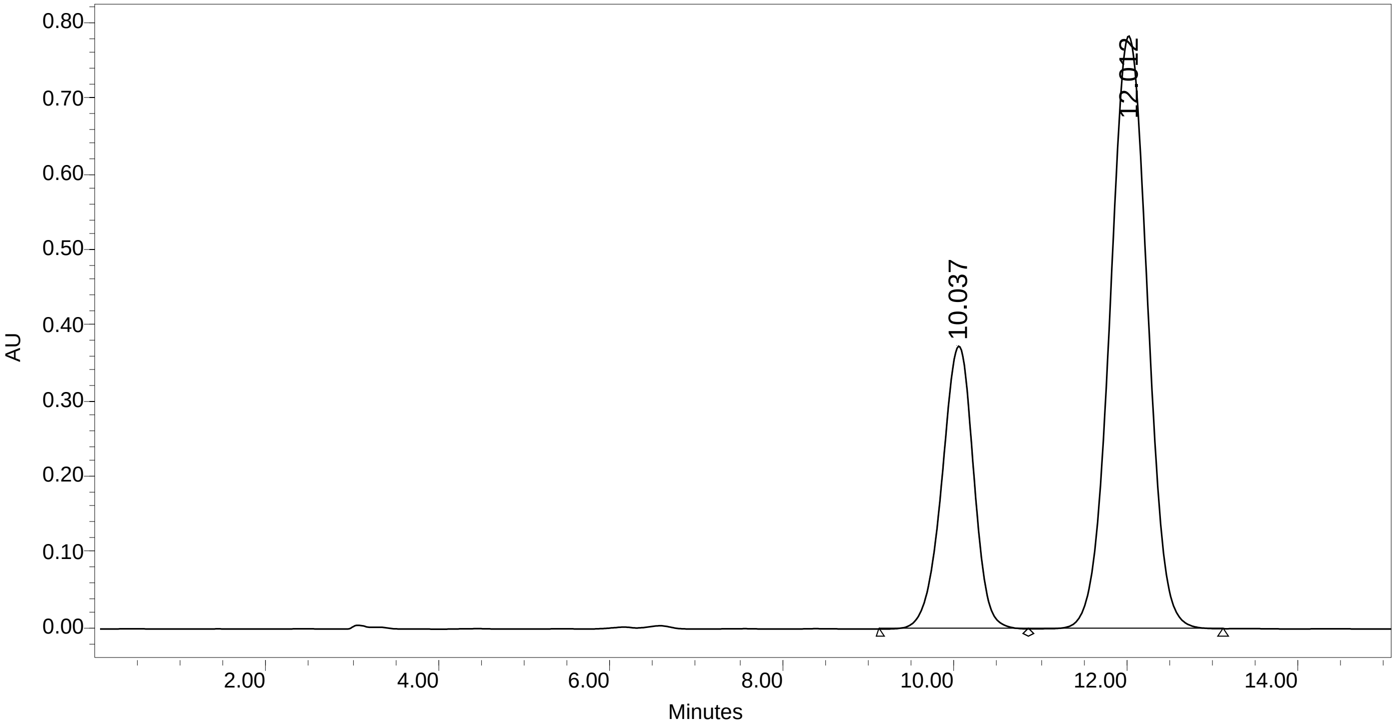

	RT (min)	Area (µV*sec)	% Area	Height (µV)	% Height
1	10.037	9597223	28.93	373760	32.29
2	12.012	23577747	71.07	783586	67.71
